# Deciphering Molecular Host-Pathogen Interactions During *Ramularia Collo-Cygni* Infection on Barley

**DOI:** 10.3389/fpls.2021.747661

**Published:** 2021-10-22

**Authors:** René Lemcke, Elisabet Sjökvist, Stefano Visentin, Manoj Kamble, Euan K. James, Rasmus Hjørtshøj, Kathryn M. Wright, Anna Avrova, Adrian C. Newton, Neil D. Havis, Simona Radutoiu, Michael F. Lyngkjær

**Affiliations:** ^1^Department of Plant and Environmental Sciences, Copenhagen University, Frederiksberg, Denmark; ^2^Crop and Soils Systems, Scotland’s Rural College, Edinburgh, United Kingdom; ^3^Institute of Evolutionary Biology, The University of Edinburgh, Edinburgh, United Kingdom; ^4^Ecological Sciences, The James Hutton Institute, Invergowrie, United Kingdom; ^5^Cell and Molecular Sciences, The James Hutton Institute, Invergowrie, United Kingdom; ^6^Department of Molecular Biology and Genetics, Aarhus University, Aarhus, Denmark; ^7^Sejet Plant Breeding, Horsens, Denmark

**Keywords:** Transcriptome (RNA-seq), Ramularia, *Hordeum vulgare*, metabolite responses, Pathogen response pathways, host defense, fungal pathogen, transmission electron microscopy

## Abstract

*Ramularia collo-cygni* is the causal agent of Ramularia leaf spot disease (RLS) on barley and became, during the recent decades, an increasing threat for farmers across the world. Here, we analyze morphological, transcriptional, and metabolic responses of two barley cultivars having contrasting tolerance to RLS, when infected by an aggressive or mild *R. collo-cygni* isolate. We found that fungal biomass in leaves of the two cultivars does not correlate with their tolerance to RLS, and both cultivars displayed cell wall reinforcement at the point of contact with the fungal hyphae. Comparative transcriptome analysis identified that the largest transcriptional differences between cultivars are at the early stages of fungal colonization with differential expression of kinases, calmodulins, and defense proteins. Weighted gene co-expression network analysis identified modules of co-expressed genes, and hub genes important for cultivar responses to the two *R. collo-cygni* isolates. Metabolite analyses of the same leaves identified defense compounds such as *p*-CHDA and serotonin, correlating with responses observed at transcriptome and morphological level. Together these all-round responses of barley to *R. collo-cygni* provide molecular tools for further development of genetic and physiological markers that may be tested for improving tolerance of barley to this fungal pathogen.

## Introduction

*Ramularia collo-cygni* (*Rcc*) is the fungus that induces Ramularia leaf spot (RLS) disease in barley (*Hordeum vulgare*) which results in significant losses of grain yield and quality (Sutton and Waller, 1988). RLS is considered as an emerging disease which has been reported only in recent years as a threat to barley cultivations in Europe and most other temperate regions (Walters et al., 2008). The life-style of *Rcc* is defined by prolonged endophytic, symptomless growth within its host, followed by a rapid switch to pathogenic interaction which leads to RLS symptom development late in the growing season, usually post-anthesis during grain filling. Interestingly, *Rcc* can complete its life cycle symptomless on a range of hosts, including barley, suggesting a partially endophytic life style (Havis et al., 2014). Typical RLS symptoms are described initially as pepper-spot necrotic lesions, loosely resembling physiological leaf spots (Havis et al., 2015). It has been shown that the fungus can spread from generation to generation *via* seed transmission and by wind and splash-dispersed spores from infected leaves with sporulating conidiophores (Walters et al., 2008; Stabentheiner et al., 2009). Mycelial growth from spores deposited on the leaves detects stomatal openings and penetrates into the stomatal cavity from where colonization of the apoplastic area of the mesophyll follows (Thirugnanasambandam et al., 2011). However, detailed morphological responses of host cells to *Rcc* infection have not been described.

Pathogen-host communication at a molecular level plays an important role during the establishment of their interplay. Typically, fungal colonization of the plant’s surface or apoplast is detected by specific membrane-anchored receptors (pathogen-recognition receptors, PRRs) that recognize pathogen-associated molecular patterns (PAMPs). This molecular interaction evokes, in the case of a pathogenic colonization, a PAMP-triggered immune (PTI) response (Jones and Dangl, 2006; Choi and Klessig, 2016; Zipfel and Oldroyd, 2017). The pathogen is able to counteract this host defense with specific secreted effectors. In turn, the plant senses the impaired PTI and reacts with effector-triggered immunity (ETI), by means of receptors recognizing these effectors or due to their activity on host components (Jones and Dangl, 2006; Cook et al., 2015). This interplay between host and fungal responses leads to complex changes in the host’s transcriptome, metabolome, and proteome (Hurley et al., 2014; Bigeard et al., 2015; Li et al., 2016). Time-course investigation of host and fungal transcriptional response provided evidence for the activation of special secondary metabolite production and transport, as flavonoid-specific genes and an array of metabolite transporter were found to be continuously activated. Phenolamides, especially accumulation of *p*-coumaroyl-agmatine (*p*-CA) and its hydroxylated forms *p*-coumaroylhydroxyagmatin (*p*-CHA) and p-coumaroyl-hydroxydehydroagmatin (*p*-CHDA), have been linked to resistance responses against several pathogens in barley (Bollina et al., 2010; Mikkelsen et al., 2015). These compounds are known not only for their antifungal properties but also for cell wall fortification *via* lignification (von Röpenack et al., 1998; Jansen et al., 2005). Similarly, serotonin and conjugates may be involved also in lignification and hence cell-wall fortification (Kanjanaphachoat et al., 2012; Hayashi et al., 2016; Ishihara et al., 2017). Furthermore, serotonin and its dimerized form have been shown to inhibit different *Candida* and *Aspergillus* species *in vitro*, indicating a potential direct antifungal function (Lass-Florl et al., 2002; Lass-Florl et al., 2003).

RLS is a relatively new disease in barley cultivation, and the underlying molecular responses mounted by different barley genotypes in the presence of different *Rcc* isolates during colonization and symptom development are unknown. A general field study on a range of spring and winter barley cultivars failed to reveal any genotypes with general resistance against *Rcc*, however a robust quantitative resistance to RLS has been shown for selected varieties under different field conditions (Havis et al., 2015; Hoheneder et al., 2021). An additional study identified the barley *Mlo* gene as a molecular factor influencing the *Rcc*-barley interaction (McGrann et al., 2014). Typically, mutations of *Mlo* lead to broad resistance against the biotrophic fungal pathogen powdery mildew (*Blumeria graminis f.sp. hordei*) and recessive *Mlo* alleles are present in most European barley breeding programs (Acevedo-Garcia et al., 2014). However, these mutations have been shown to be ineffective against infection with other barley pathogens, or may even increase susceptibility to necrotrophic barley pathogens like *Magnaporthe grisea* (blast), *Cochliobolus sativus* (spot blotch) or *Fusarium graminearum* (head blight) (Jarosch et al., 1999; Kumar et al., 2001; Jansen et al., 2005; McGrann et al., 2014). A previous study on *Ramularia*-barley interaction revealed a marginal up-regulation of the wild-type *Mlo* gene during infection by the aggressive DK05 isolate and suggested that MLO is a possible target for a fungal effector (Sjökvist et al., 2019).

Here we show that the apoplastic infection of barley by *R. collo-cygni* is accompanied by structural changes in the cell wall of the host that are substantiated by changes identified from comparative transcriptome and metabolite analysis. We performed a detailed molecular investigation of responses induced by two barley cultivars identified as tolerant or sensitive to RLS infection by two fungal isolates that trigger different levels of host response. Transcriptome and metabolome responses were monitored at different time-points post-infection in order to capture host reaction to the symptomless foliar and subsequent apoplastic colonization and severe symptom development. Electron microscopy of symptom-developing leaves was used to identify morphological responses at the ultrastructure level. Together, these provide a comprehensive analysis of responses of the barley host, identifying key molecular events at different stages of *Rcc* colonization. These responses can be further dissected in more detailed molecular analyses of individual pathways and extended to larger collections of barley cultivars to fully correlate with increased tolerance to RLS.

## Materials and Methods

### Barley Cultivars and Fungal Isolates

Two different barley (*Hordeum vulgare*) cultivars, Fairytale (Sejet Planteforaedling, Denmark) and NFC Tipple (Syngenta Seeds, United Kingdom) (Tipple, henceforth). The cultivars developed in previous field trials either strong or subtle disease symptoms, respectively. Further, two contrasting *Rcc* isolates DK05 and NZ11 were used for analysis. Isolate DK05 was isolated from the highly susceptible spring barley cv Braemar in Denmark in 2005. The *R. collo-cygni* isolate NZ11 was isolated 2011 in New Zealand (Templeton) from the spring barley cv Doyen, which is susceptible to Ramularia leaf spot (Oxley and Havis, 2010; McGrann et al., 2016).

### Sampling and Preparation for Transmission Electron Microscopy

Leaf samples for TEM investigations were prepared by inoculating a fully developed second leaf of barley cv Fairytale with a *Rcc* mycelial suspension. GFP-labeled DK05 and NZ11 isolates have been used for visualizing the infected leaf areas necessary for defining the samples for transmitted electron microscopy (TEM) (Thirugnanasambandam et al., 2011; Visentin, 2019). The mycelial suspension was prepared by homogenization of a 2-week-old PDB *Rcc* culture incubated on a SSL1 orbital shaker (Stuart^®^) at 120 rpm at 18°C in the dark. Mycelia was collected by centrifugation at 1000 *g* for 10 min and resuspended in 20 mL SDW. The suspension was then homogenized for 60 s at maximum speed in a column mixer and filtered through a 100 μm nylon mesh. The flow-through was then centrifuged at 1000 *g* for 10 min to collect the mycelial fragments. The pellet obtained was resuspended in 10 mL 0.05% Tween20 solution. The resulting homogenized mycelial solution was assessed with the use of a hemocytometer for the presence of viable and active hyphae (without Rubellin) and bacterial contamination. Prior to inoculation, barley leaves were gently stroked with a clean brush to enhance infection by disrupting the leaf surface waxes. Second forming leaves were inoculated with a 2 μL drop of inoculum and incubated in a controlled environment microclima 1000 (Snijders Scientific) cabinet at 18°C providing light for 16 h a day, and an 8 h night at 12°C. Samples were harvested at 21 and 27 days post infection (dpi) and fixed in 2.5% paraformaldehyde (PFA) and 0.25% glutaraldehyde in 0.1 M sodium cacodylate buffer pH 7.0, degassed under vacuum until sample sinking and incubated at 4°C for 72 h. Fixed samples were washed in 0.1 M sodium cacodylate buffer pH 7.0 twice, dehydrated in 99.97% ethanol for 20 min and soaked in hard grade acrylic LR White Resin (Agar Scientific) on a Rotator Type N (TAAB) at 2 rpm. The samples were embedded by hardening the resin at 65°C for 48 h and subsequently sectioned into 80 nm ultra-thin sections using a Leica Ultracut UCT Ultramicrotome. Sections were collected on 200 mesh hexagonal nickel grids (Smethurst High-Light Ltd, agar scientific, G2450N) previously coated in pyroxylin. Prior to labeling, the sections were blocked for 60 min in 1% bovine serum albumin (BSA) in 1% PBS buffer pH 7 (IGL buffer) to reduce antibody non-specific binding. Fungal cell walls components were labeled with 10 nm wheat germ agglutinin (WGA) colloidal gold conjugated *Triticum vulgare* (EY laboratories^©^) diluted 1:100 in IGL buffer. Finally, labeled sections were washed twice for 5 min in IGL buffer and five times for 1 min in sterile double-distilled water (ddH_2_0). Sections were imaged using a JEOL JEM1400 transmission electron microscope.

### RNA-Seq Analysis of Infected and Uninfected Samples

The same plant and fungal growth conditions, fungal inoculation procedure and DNA/RNA extraction methods were used as described previously (Sjökvist et al., 2019). A detailed description is included in the Supplementary Material. The two cultivars and isolates have been tested in parallel at the analyzed time points, and the Fairytale-DK05 interaction described before (Sjökvist et al., 2019) is therefore included here as reference. The RNA isolation, sequencing details, read mapping and read counting was performed as presented in Sjökvist et al. (2019). A detailed description of RNAseq read mapping and counting as well as the procedure of the TPM calculations, differential gene expression and GO enrichment analysis can be found in the Supplementary Material.

### Co-expression Analysis, Hub-Gene Identification, and Network Analysis

Co-expression analysis was performed using the WGCNA package in R under the guidelines of the published tutorials (Zhang and Horvath, 2005; Langfelder and Horvath, 2008). HT-seq counts were imported to R and transformed to a DESeq dataset (Love et al., 2014), and subsequently an estimation of size factors and dispersion and GLM fitting was performed. Data were transformed into DESeq2 dataset in order to perform the in-build variance stabilizing transformation, a normalization technique for the count values before the Weighted Correlation Network Analysis (WGCNA). Only genes with recorded counts of 10 and higher in at least 9 samples were included in the analysis. Moreover, genes were tested for their coefficient of variation (COV) to exclude genes with low variation. Genes with a COV below 0.15 were excluded from the analysis as well, leaving a dataset including 5,583 genes for the co-expression analysis. Further WGCNA analysis was performed using default settings with minor changes. We used a soft power of 5 for the calculation of network adjacency of gene counts and the topological overlap matrix (TOM). Subsequent clustering of modules was completed with a minimum module size of at least 15 genes and a dynamic tree cut of 0.95 for the adaptive branch pruning of the hierarchical cluster dendrogram. In order to combine modules that are too close, we merged close modules at a maximum dissimilarity of 0.1. These settings merged the 5,583 selected genes into 21 modules. For each module, 20 hub-genes were identified based on their module membership. For the network analysis, the highest absolute values of the weights for the connections between those genes in the TOM and transformed them to edge and node data needed for the network analysis. Network visualization was done with the Cytoscape platform.

### Sample Preparation for Metabolite Extraction, Instrumentation, and Compound Identification

At each time point of sampling three approximately 2 cm long leaf pieces were cut from different plants, pooled and weighed as one sample and subsequently snap frozen in liquid nitrogen upon sampling and stored at -80°C until extraction. Leaf tissue was ground constantly frozen using a tissue lyser for 2 min at the highest frequency (30,000 Hz). The metabolite extraction protocol was performed as previously described by Mikkelsen et al. (2015) with minor modifications. In brief, polar metabolites from the frozen leaf powder were extracted by adding 500 μl 85% MeOH- HPLC grade (v/v) containing 250 μM Amygdalin as an internal standard. The tubes were locked with security caps to prevent MeOH from evaporating. Immediately hereafter the tubes were placed in a Thermo block pre-heated at 100°C and boiled for 5 min. The extracts were kept on ice and diluted 1 in 5 with ddH_2_O and filtered through an Anopore 0.45 μm filter (Whatman) before analysis. Metabolite analysis was performed 3–5 days after extraction.

Analytical LC–MS was performed using an Agilent 1100 Series LC (Agilent Technologies) coupled to a Bruker HCT-Ultra ion trap mass spectrometer (Bruker Daltonics). A Zorbax SB-C18 column (Agilent; 1.8 l M, 2.150 mm) was used at a flow rate of 0.2 mL min^–1^, and the oven temperature was maintained at 35°C. The mobile phases were as follows: A, water with 0.1% (v/v) HCOOH and 50 l M NaCl; and B, acetonitrile with 0.1% (v/v) HCOOH. The gradient program was as follows: 0–0.5 min, isocratic 2% B; 0.5–7.5 min, linear gradient 2–40% B; 7.5–8.5 min, linear gradient 40–90% B; 8.5–11.5 isocratic 90% B; 11.60–17 min, isocratic 2% B. The flow rate was increased to 0.3 mL min^–1^ in the interval 11.2–13.5 min. The mass spectrometer was run in positive electrospray mode. The dry gas flow was 10 L min^–1^ at 365°C. The mass range m/z 100–800 was acquired. The data were analyzed using Bruker Data Analysis v.4.0. Integrated extracted ion chromatograms peak areas were used as estimates for compound concentrations.

Major peaks in the total ion chromatogram from healthy and infected leaves at different time points were identified based on their fragmentation patterns as described by Mikkelsen et al. (2015). The focus of the search was soluble phenolic compounds previously identified in barley leaves and peaks that accumulate differentially in control versus infected samples.

## Results

### *Ramularia Collo-Cygni* Colonization Leads to Changes in the Cell Wall Structure of Barley

**FIGURE 1:**
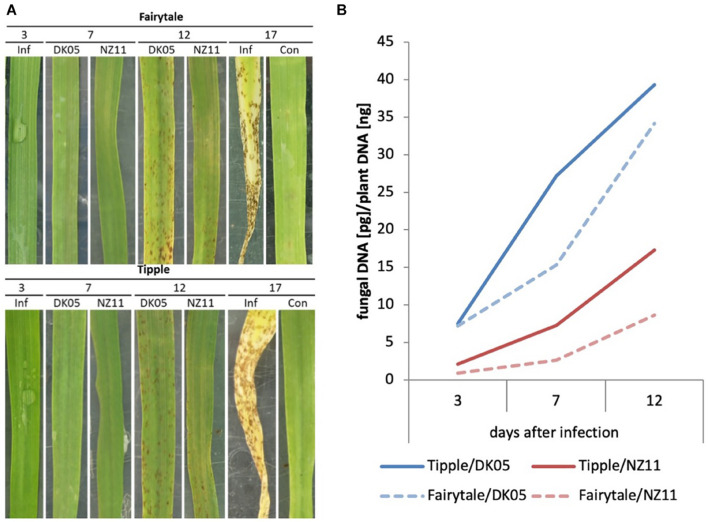
Ramularia leaf spot development and Rcc biomass development of barley leaves (*Hordeum vulgar*e L) on cv Tipple and cv Fairytale with two different isolates (NZ11 and DK05). **(A)** Development of necrotic leaf spots throughout the monitored time course experiment at 3, 7, 12, and 17 days post inoculation (dpi). At 3 dpi an exemplary picture of an infected (Inf) but symptomless leaf was included. This phenotype was seen for both isolates. 17 dpi was included to show the complete collapse of an infected host leaf in comparison to an mock-inoculated leaf (Con), indicating accelerated senescence due to infection. **(B)** Relative content of Rcc DNA in infected samples at 3, 7, and 12 days post infection (dpi). Two different isolates [Rcc DK05 (aggressive), Rcc NZ11 (mild)] were monitored on two different barley cultivars (cv Tipple and cv Fairytale).

**FIGURE 2:**
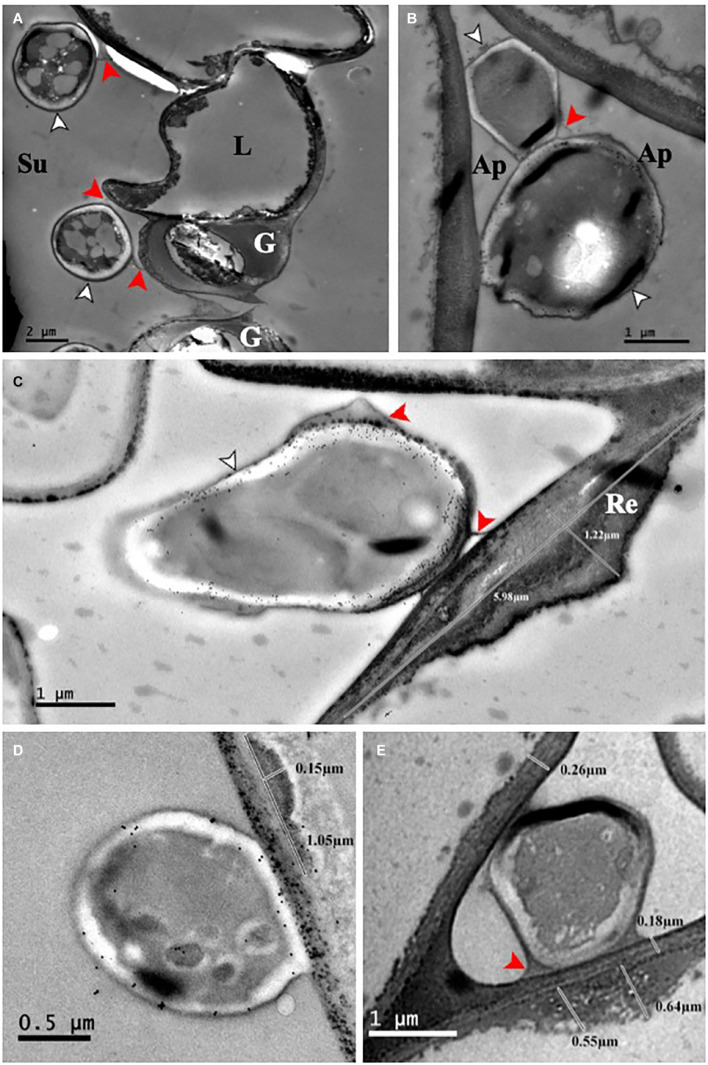
*Ramularia collo-cygni* – barley (cv Fairytale) adherence and plant response. **(A)** NZ11-GFP, 21 dpi. In the substomatal cavity, the hyphae adhere to the epidermal and guard cells through an ECM. **(B)** DK05-GFP, 27 dpi. Infecting hyphae growing intercellularly directly adhere to the cell wall. **(C)** DK05-GFP 21 dpi. The ECM protrudes from the fungus toward the plant cell wall. At the site of contact between the fungus and the host cell, a plant cell wall reinforcement is visible. **(D)** DK05-GFP 27 dpi WGA colloidal gold labeled. **(E)** DK05-GFP 21 dpi unlabeled. Ap, apoplast; G, guard cell; L, lateral cell; Re, cell wall reinforcement; Su, substomatal cavity; Hyphae, white arrowhead; ECM, red arrowheads.

In this study we used two barley cultivars, Fairytale and Tipple, that in previous field trials developed either strong or subtle RLS disease symptoms, respectively. For infections we utilized *Rcc* isolates DK05 and NZ11 that induced strong or mild RLS symptoms, respectively, under test conditions (Figure 1 and Supplementary Figure 19). We previously described the response of Fairytale to DK05 infections at three time points post infection (3, 7, and 12 dpi) (Sjökvist et al., 2019), and integrated this dataset for computational analyses and comparability with the additional interactions presented here. Comparative analysis to Tipple identified the latter developed comparably less RLS disease symptoms than Fairytale. Likewise, *Rcc* isolate DK05 caused more RLS disease symptoms than NZ11, confirming previous indications based on field observations. We quantified fungal growth by qPCR fungal DNA analysis. These results showed a faster colonization of DK05 in both barley cultivars compared to NZ11 (Figure 1B). However, overall *Rcc*-DNA levels were lower in Fairytale than Tipple indicating no correlation between RLS disease symptoms and the amount of fungal biomass present (Figures 1A,B). To investigate the responses of barley cultivars to apoplastic infection of *Rcc* in detail, electron micrographs were produced of infected, symptom-developing barley leaves. *Rcc* hyphae were found exclusively in the sub-stomatal cavity (Figure 2A) and in the apoplastic spaces between mesophyll cells (Figure 2B). In most cases, *Rcc* hyphae adhered to plant cell walls through an extracellular matrix (ECM) (Figure 2). This ECM appeared to be of fungal origin, protruding from the hyphae toward the plant cell wall. In response to contact with hyphae or the ECM, the plant cell produced wall appositions at the contact sites. This was observed as secondary wall reinforcement structures measuring up to 1.22 μm in thickness and 6 μm in length (Figures 2C–E). No signs of hyphal penetration of barley host cells were observed. Together, our observations revealed large ultrastructural changes in the host and in the fungus during pathogenic stage of *Rcc*-barley infection.

### Differential Gene Expression – General *Ramularia Collo-Cygni* Responsive Regulatory Networks

**FIGURE 3:**
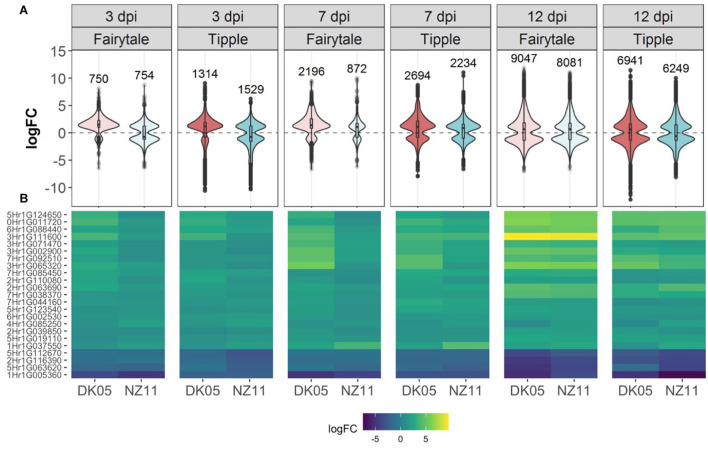
Differentially expressed (DEG) barley genes during Ramularia leaf spot (RLS) disease progression. **(A)** Violin plots with included box-whisker plots depicting the general trend of up- and down- regulation during RLS progression on barley (*Hordeum vulgare L*) cv Fairytale and cv Tipple during foliar infection with *Rcc* isolates DK05 and NZ11. **(B)** Heatmap of differentially expressed (logFC) core genes. Gene IDs are shortened (HORVU removed).

Leaf samples from the two cultivars infected by one of the two isolates have been analyzed for transcriptional responses at 3, 7, and 12 dpi. An overall view of the number of differentially expressed genes (DEG) in infected versus control, untreated samples of the same age, revealed that both cultivars had an increasing number of DEGs with the increasing number of days of interactions with the *Rcc* isolates (Table 1). This reflects the accelerated host response to the continuous fungal colonization and increase in fungal biomass (Figure 1). Across the three time points and interaction with both isolates, Tipple had fewer DEGs compared to Fairytale (Figure 3 and Table 1). Furthermore, NZ11 induced fewer DEGs compared to DK05 in both cultivars. We identified only 23 genes (Table 2) continuously regulated at all three time points and in all four interactions. Irrespective of the inoculum, we found that Tipple had twice as many DEGs as Fairytale at 3 dpi, while at 12 dpi Fairytale had substantially more DEGs than Tipple. At 7 dpi, Tipple and Fairytale responded with a similar number of DEGs to DK05, but differed greatly in response to NZ11, with Fairytale having only about a third of the number of DEGs compared to Tipple. This general view of transcript regulations illustrates that responses of barley to *Rcc* are largely time-, cultivar-, and isolate-dependent.

**TABLE 1:**
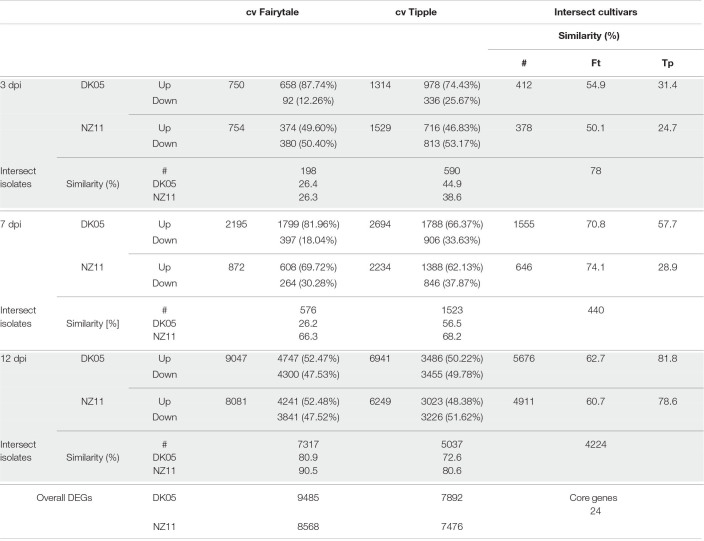
Differentially expressed gene number in different treatments and time points.

*The number of DEGs at the different time points during infections with Rcc isolates DK05 and NZ11 on barley cultivars Fairytale and Tipple are presented in italic in corresponding letters. Numbers for up- and down-regulation are presented in raw numbers and in percentages to the overall number. DEGs in intersects are presented in raw numbers and in percentage of the corresponding treatment at the time.*

**TABLE 2:**
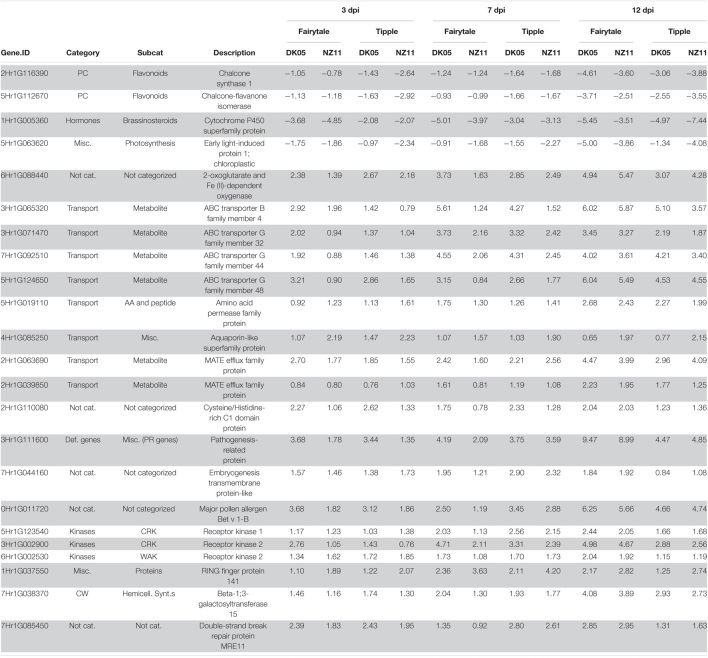
Differentially expressed barley genes regulated at all treatments and all time point during *Ramularia collo-cygni* infection.

*Values shown represent logFC comparing an infected sample against the corresponding control sample. All values genes depicted had a FDR value < 0.05 in all treatments. PC, phenolic compounds; Misc., miscellaneous; Not cat., not categorized; Def. genes, defense genes; CW, cell-wall genes; AA, amino acid; CRK, cysteine rich kinase; WAK, wall associative kinase, PR, pathogenesis related genes; Hemicell. Synt., hemicellulose synthesis.*

### Fairytale and Tipple Differ in the Expression Level of a Large Number of Genes

The two cultivars were chosen based on their differential response to *Rcc* (Figure 1), and a possible explanation for this response might reside in native, differential gene expression. To test this, we have searched for genes with a significantly different expression level (logFC > 2 or < −2, FDR lower than 0.05) in the control samples of the two cultivars, at any of the analyzed time points. This identified 461genes of which only 173 were found either non-regulated or not significantly (FDR higher than 0.05) regulated by *Rcc*. The majority of these 173 genes (*n* = 74) encode for non-categorized proteins, but also defense genes (*n* = 17, of which three CC-NBS-LRR proteins), kinases (*n* = 26, of which seven LRR receptor-like kinases), transcription factors (*n* = 5), and transporters (*n* = 13). (Supplementary Table 3 and Supplementary Figures 3, 4). Interestingly, most of them (*n* = 121) have a higher level of expression in Tipple, but we found six genes coding for defense proteins (two CC-NBS-LRR and four PR) and transporters for sugar, phosphate, oligopeptide, and nitrate to have a higher level of expression in Fairytale, indicating that the apoplastic environment in the two cultivars might be different, and with consequences for *Rcc* growth and responses. Among the 288 genes differently expressed between cultivars and significantly regulated by *Rcc* we found cell wall related proteins (*n* = 14; expansins, and xyloglucan hydrolases) having a higher level of expression in control Tipple at 7 dpi, and to be down-regulated in both cultivars during infection by *Rcc*, particularly at 7 dpi and by DK05. A large number of kinases (*n* = 50), of which many receptors (*n* = 36), differ in expression between Fairytale and Tipple, majority of them having higher expression levels in Tipple, and being up-regulated by *Rcc*, some already from 3 dpi. Similarly regulated we found 14 genes coding for WRKY, NAC, or bHLH-type transcription factors. Defense genes (*n* = 23) coding for PR proteins, wound-induced proteins, and chitinases were also among them. Four genes coding for UDP-glycosyltransferases also had higher expression in Tipple and were induced in both cultivars by *Rcc* at 3 dpi. Interestingly, defense genes with higher levels of expression in Fairytale are not up-regulated by *Rcc* in this cultivar, but in Tipple are induced at 7 dpi. By contrast, defense genes that have a higher expression level in Tipple are up-regulated by *Rcc* at all time points in both cultivars infected with DK05 and from 7 dpi when infected by NZ11. This indicates that signaling for up-regulation of these genes in the two cultivars differs and this might contribute to the early differential response to *Rcc*. A further analysis of the 461 genes with differential expression between cultivars showed that some of these are tandemly arranged and encode receptor kinases (HORVU1Hr1G002590, HORVU1Hr1G002600, HORVU4Hr1G000030, and HORVU 4Hr1G000040), NAC transcription factors (HORVU5Hr1G0 99460 and HORVU5Hr1G099470), and PR proteins (HORVU7Hr1G115990 and HORVU7Hr1G116000) that could be considered as candidate molecular markers present in barley cultivars with increased tolerance to *Rcc*.

### Barley Responses to *Ramularia Collo-Cygni* at 3 Days Post Infection Are Both Cultivar and Isolate Dependent

**FIGURE 4:**
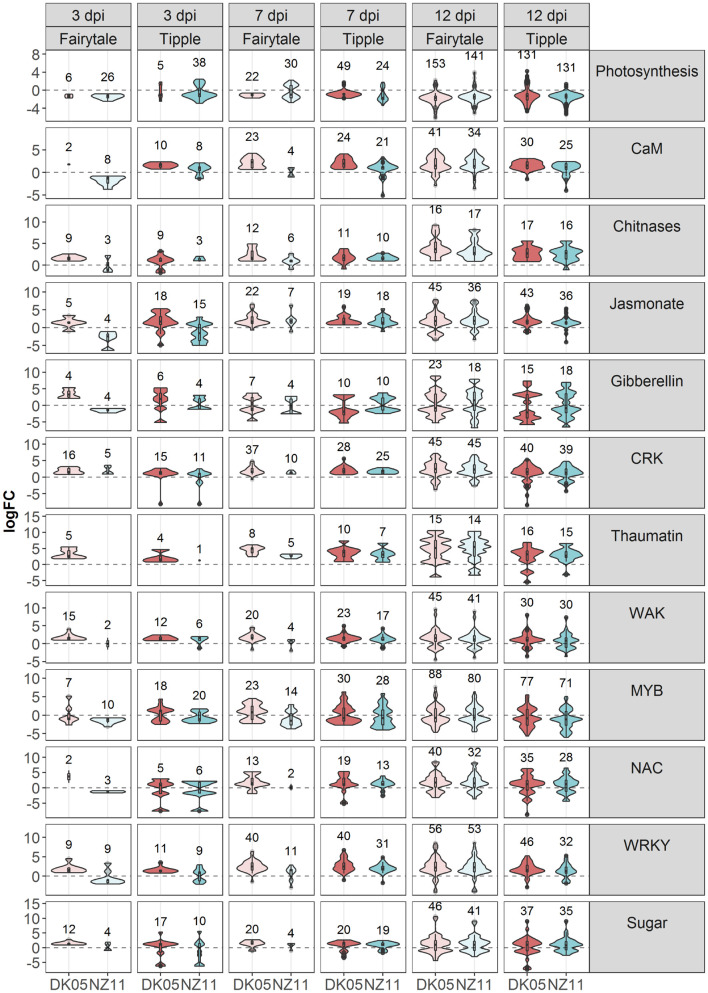
V| iolin plots depicting differentially expressed (DEG) barley genes on sub-categories of molecular gene functions affected by *Rcc* colonization and disease progression. Violin plots with included box-whisker plots depicting the general trend of up and down regulation during RLS progression on barley (*Hordeum vulgare L*) cv Fairytale and cv Tipple during foliar infection with *Rcc* isolates DK05 and NZ11.

A substantial number of genes were differentially regulated by *Rcc* infection (logFC in *Rcc* versus mock-treated samples) at 3 dpi, but we found little overlap (78 of 2785 DEGs) in the four analyzed interactions (Table 1), suggesting that the initial response to *Rcc* colonization depends both on the barley host and the *Rcc* isolate. Among the 78 DEGs commonly regulated we identified the 23 genes presented in Figure 3A and Table 2, as well as six genes involved in transport, four transcription factors, five genes involved in phytohormone signaling (esp. ethylene), five kinases and three oxidoreductases (Supplementary Table 1). Analysis of strong down-regulated DEGs (logFC < −5) in Tipple showed that both *Rcc* isolates induced a reduced expression of the same approximately 100 genes (Figure 4) encoding disease resistance proteins, transcription factors, receptors, and unknown proteins (Supplementary Table 1). Importantly, none of the genes were differentially expressed in Fairytale at this time point, and thereby represent unique responses induced by Tipple in response to *Rcc* infection. A similar analysis identified only 6 genes specifically up-regulated in Tipple, indicating that at this early time point the specific reactions of the tolerant cultivar was primarily reflected in gene down-regulation.

To investigate the common response to infection by the same *Rcc* isolate we identified overlapping DEGs for DK05 (*n* = 412) or NZ11 (*n* = 378) infected samples (Table 1). A common pattern for both cultivars was the prevalence of up-regulating genes during DK05 infection (658 up- vs. 92 down-regulated), compared to a more balanced response during NZ11 infection (374 up- vs. 380 down-regulated, Table 1). Most of the DEGs up-regulated by DK05 in both cultivars encoded receptors and defense related proteins, such as PR-proteins and chitinases. Genes down-regulated by NZ11 in both cultivars encoded enzymes for defense and photosynthesis (Supplementary Figure 6). Together, this indicates that the two isolates elicited specific regulation of differential defense responses at this early time point of the interaction.

### 7 Days Post Infection – NZ11 Induces a Limited Transcriptional Response in Fairytale During Apoplast Colonization

Comparative host responses in the four interactions, at 7 dpi showed, with one exception, Fairytale infected by NZ11, a large overlap in responses (Table 1). In Fairytale only a third of DEGs identified in DK05-infected samples were also regulated by NZ11. Among these, we found genes involved in cell wall modifications and defense responses to be up-regulated, and photosynthesis associated genes to be down-regulated (Supplementary Figure 5). In general, the genes found to be differentially regulated at 7 dpi had the same molecular functions and the same direction of regulation as observed at 3 dpi (Figure 3 and Table 1), with the exception of genes associated with defense processes that were found up-regulated in Fairytale and down-regulated in Tipple. These included genes coding for cell wall modification enzymes, transmembrane transport, and in biosynthesis of phenylpropanoids, flavonoids and ethylene pathway (Figure 3 and Supplementary Figure 11). We found that DK05 infection of the same host was associated with a larger number of DEGs compared to NZ11, these encoding proteins involved in defense such as NAC, MYB and WRKY transcription factors, LRR-domain proteins, receptor-like protein kinases and biosynthesis of phenylpropanoids, flavonoids, and ethylene (Supplementary Figures 8, 9, 13).

### 12 Days Post Infection – The Phase of Pathogenic Attack and Severe Disease Responses

The overall responses observed at the time when disease symptoms manifest on the leaves was that, compared to earlier time points, a larger number of barley genes were regulated in all four interactions, (Table 1). Overall, Fairytale had a stronger response at 12 dpi (about 2,000 more DEGs), but only 60% of these genes were also found differentially regulated in Tipple (Table 1) due to a lower number of overall regulated genes in Tipple. This differential regulation was primarily observed for genes involved in defense (chitinases, dirigent-like, and NB-ARC domain coding genes), phytohormone signaling and biosynthesis (JA, gibberellin and ethylene), cell wall processes (pectin esterases), kinases (LysM, Thaumatin), phenolic compound synthesis (simple phenols) and oxidoreductases (glutathione S-transferases and peroxidases) (Figure 4 and Supplementary Figures 10, 11). DK05 infection of Tipple led to strong down-regulation (logFC < −5) of genes (*n* = 76) that were not found in any of the other three interactions. These included LRR-domain encoding defense genes, as well as cysteine-rich kinases, thaumatin receptor-like protein kinases and NAC transcription factors, sugar transporters, and genes involved in the production of phenylpropanoids and cell-wall degradation (Figure 4). These indicate that even if most of the DEGs are similarly regulated in the two cultivars, there are clear differences between responses induced by Tipple and Fairytale also at this late stage of infection.

### Co-expression Clustering and Corresponding Hub Genes Reveal a Heterogeneous Collection of Stress-Induced Response Patterns

**FIGURE 5:**
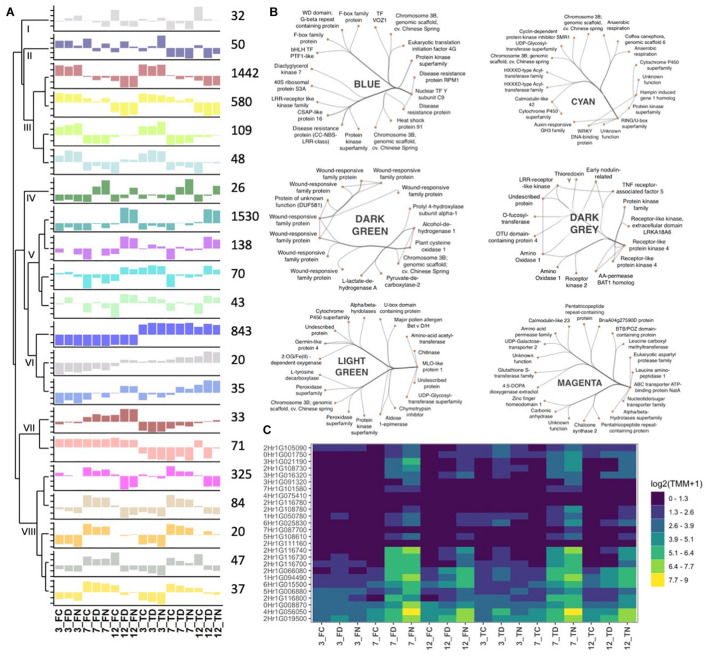
Eigenvalue barplot for co-expression analysis. **(A)** Each plot depicts the average eigengene values across the biological replicates for all interactions at each time point. Dendrogram sorts modules according to their hierarchical clustering. Numbers close to the module dependent bar plots show the number of gene included in each module. The names of the modules are as following: (I) gray; (II) midnightblue; (III) brown, yellow, greenyellow, and lightcyan, (IV) darkgreen; (V) darkturquoise, purple, cyan, and lightgreen; (VI) blue, darkgray, and royalblue; (VII) darkred and salmon; (VIII) magenta, tan, orange, gray60, and lightyellow. **(B)** Gene networks for selected modules for 20 highest hub gene connections. **(C)** Expression levels of genes (TMM, trimmed mean of M) in the darkgreen module. Gene IDs are shortened (*HORVU* removed). F, fairytale; T, tipple; D, Ramularia isolate DK05; N, Ramularia isolate NZ11.

**FIGURE 6:**
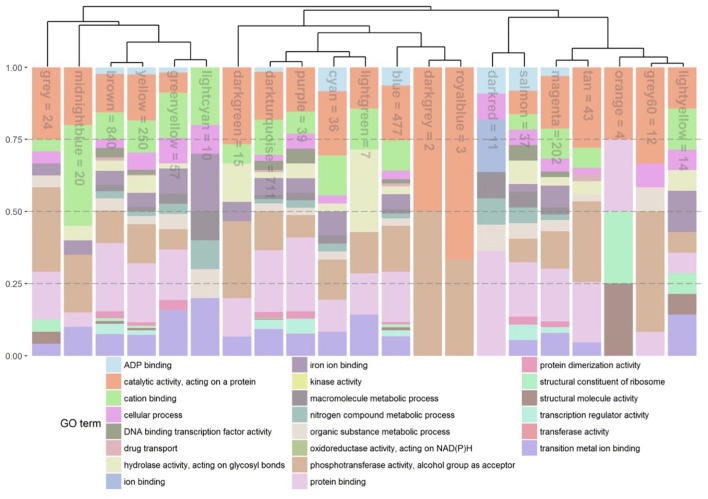
Stacked relative bar plots depicting the proportions of significant enriched GO terms in the identified gene co-expression modules. GO-terms were filtered and superior GO-terms [as biological process (bp), cellular component (cc), and molecular function (mf)] were removed. Gene modules and associated bars are sorted by a hierarchical clustering represent by the dendrogramm. Name of module is presented inside the bar. The number corresponds to the number of genes left inside the module after removing the main GO terms.

To further dissect *Rcc*-induced transcriptional responses of barley, we grouped genes that exhibited similar expression profiles across our samples into response modules. For this, we performed a weighted gene correlation network analysis (WGCNA) on 5,583 genes (see Materials and Methods for details) (Supplementary Table 2) (Langfelder and Horvath, 2008). Based on the constructed network topology, we identified 21 stress-response modules and their highly intra-connected hub genes (Figure 5A). The modules clustered in two main clades organized into 8 subgroups whose formation was associated with enrichment of condition-specific biological processes (Figure 6). The first clade accommodates subgroups I to III with modules assembling genes that have no distinct expression pattern (*gray* = 32 genes, *midnightblue* = 50 genes) or modules with genes primarily regulated by the time point of analysis (*brown* = 1442 genes, *yellow* = 580 genes, *greenyellow* = 109 genes, and *lightcyan* = 48 genes) (Figure 5A and Table 3). Time-regulated modules were enriched in genes controlling photosynthesis, cell wall modifications, regulation of transcription, lipid biosynthesis, post-translational protein modification, signaling, transmembrane transporter activity, and cellular redox homeostasis. The second clade accommodates 5 subgroups (IV to VIII), all showing gene expression responses to *Rcc* infection (Figure 5A and Table 3). Subgroup IV contains *darkgreen* module assembling genes (*n* = 26) differently regulated by the two isolates at the 3 time points (Figure 5C). These genes are rapidly induced by DK05 at 3 dpi, by both isolates at 7 dpi, and maintain a higher level of expression in NZ11 infected samples at 12 dpi. A number of genes in this module are wound-responsive proteins and hypoxia-responsive genes. The intra-connection of hub genes in this module shows two distinct regulatory directions of “wound-responsive genes” which might be important for differentiating defense processes for NZ11 or DK05 (Figure 5B). Subgroup V consists of modules *darkturquoise* = 1530 genes, *purple* = 138 genes, *cyan* = 70 genes, and *lightgreen* = 43 genes containing genes mainly regulated in response to *Rcc* inoculation (Figure 5A and Table 3). This subgroup contains approximately 30% of the analyzed genes and was enriched in processes like response to stress, transmembrane receptor, protein kinase, chitinase, or monooxygenase activities and calcium signaling. Interestingly, the regulatory network for these modules seems to be controlled in all cases by one key hub gene (Figure 5B). The gene coding for “*MLO*-like protein 1” was a key regulatory element in the *lightgreen* module and the “RING U-box” gene in the *cyan* module. The “*MLO*-like protein 1” gene is directly connected with other defense-related genes (as chitinases, germin-like proteins and peroxidases) and with genes from primary and secondary metabolism (i.e., tyrosine decarboxylase and aldose 1-epimerase) (Figure 5B). These genes have a decreasing expression in control samples from 3 to 12 dpi, but higher expression in DK05 infected samples at 3 dpi and in DK05 or NZ11 infected samples at 7 and 12 dpi. Subgroup VI consists of the modules *blue*, *darkgray* and *royalblue* with genes (*n* = 843, 20, and 35, respectively) whose regulation is primarily associated with the cultivar, and/or their responses to *Rcc* (Figure 5A, Table 3 and Supplementary Figure 17). Importantly, this subgroup contains many defenses genes, protein kinases, receptors and transporters (Supplementary Figure 17). Among the 45 genes predicted to play a role in disease resistance that are regulated during *Rcc* infection we identified CC-NBS-LRR proteins, as well as genes coding for RGA2, RPM1, and RPP13 proteins. Transcription factors and receptor-like kinases are key features in subgroup VI modules (*blue* and *darkgray*, respectively), possibly orchestrating the defense proteins (Figure 5A and Supplementary Figure 17). Since Fairytale and Tipple differ in their tolerance to *Rcc*, genes included in this subgroup may contribute to the different responses they mount in responses to infection. Subgroup VII consists of the *dark red* and *salmon* modules containing genes (*n* = 33 and 71) with cultivar dependent expression patterns that can be regulated by *Rcc* (*darkred*) (Figure 5A). Genes involved in general metabolism and defense are present in these networks. Subgroup VIII consists of five modules (*magenta* = 325 genes, *tan* = 84 genes, *orange* = 20 genes, *gray60* = 47 genes, and *light yellow* = 37 genes) with genes regulated by *Rcc* (Figure 5A) coding for signaling proteins (cell recognition, gene regulation, kinases, transmembrane transport activity especially metal ion transport, enzyme inhibitor activity). The hub gene in the *magenta* module is an ABC-transporter binding protein connecting to other important transport processes (UDP-galactose transporter), diseases associated genes (Calmodulin-like 23) and secondary metabolism (Chalcone synthase 2). Interestingly, the regulatory network for module *orange* containing genes primarily responding to the time point of analysis that encode F-box proteins and histones, is regulated by a gene with an unknown function (Figure 5A and Supplementary Figure 18).

**TABLE 3:**
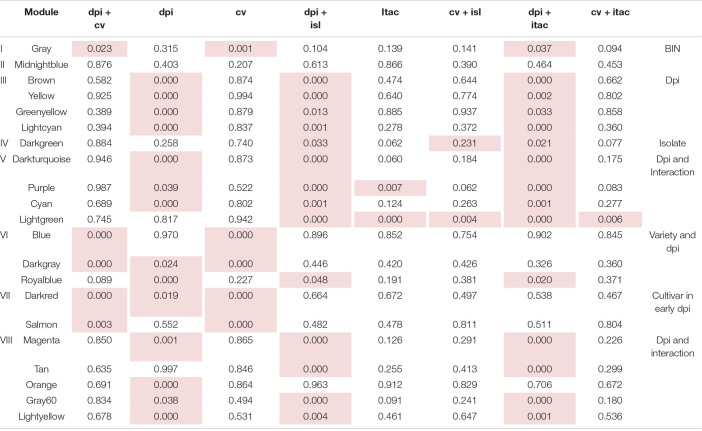
*p*-Values from GLM analysis depicting differences in between the identified co-expression gene modules.

*Column names show the factors that were compared for differences. Roman numerals are representing the sub-clusters the gene modules belong to. Clustering is based on a hierarchical clustering performed before. Last column shows the leading factor or factors separating the genes modules in the cluster from other modules. dpi, days post infection; cv, cultivar; isl, isolate; itac, interaction. Cells with red background highlight significant p-values (< 0.05) for the GLM analysis.*

### Production of Secondary Metabolites During *Ramularia collo-cygni* Infection on Barley

**FIGURE 7:**
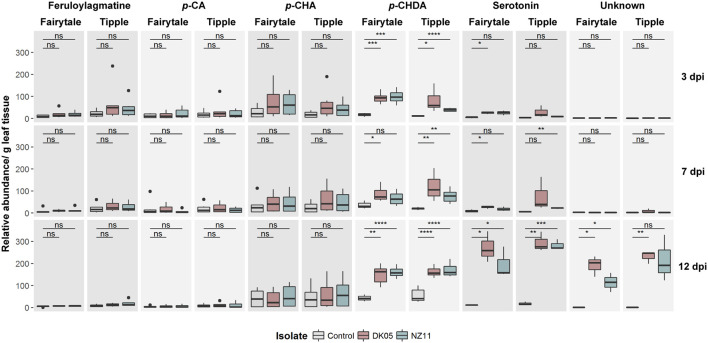
Accumulation of *Ramularia collo-cygni* (*Rcc*) responsive secondary metabolites during *Rcc* colonization in different barley cultivars (*Hordeum vulgare L*). Two different barley cultivars (cv Fairytale and cv Tipple) were monitored for disease development over a specific time course. Metabolite depicted here are responsive too *Rcc*. Two different fungal isolates [DK05 (red) and NZ11 (blue)] were used for separate infections. Control (gray) treatments were mock inoculates with water. Data shown as box plots separated by metabolites in different varieties; unpaired *t*-test; *p < 5e-2, **p < 1e-2, ***p < 5e-3, and ****p < 1e-3, ns, not significant.

In order to identify whether transcriptional changes can be monitored at the metabolite level, we performed a metabolite analysis on corresponding samples. This identified 6 metabolites that were putatively assigned to feruloylagmatine, *p*-coumaroyl-agmatine (*p*-CA), *p*-coumaroyl-hydroxyagmatine (*p*-CHA), *p*-coumaroyl-hydroxydehydroagmatine (*p*-CHDA) (phenolamides), serotonin and an unknown compound (Figure 7). The levels of feruloylagmatine, *p*-CA and *p*-CHA were stable across conditions, but a significant increase in levels of *p-*CHDA was identified in the infected leaves, at almost all time points and in all four interactions, with the exception of NZ11-infected Tipple leaves. (Figure 7). The levels of serotonin and the unknown compound increased significantly in 12 dpi*-Rcc* infected leaves from all four interactions. Biosynthesis of *p*-CA is catalyzed by an agmatine coumaroyltransferase (ACT) (von Röpenack et al., 1998; Kristensen et al., 2004; Muroi et al., 2009), but the enzymes responsible for production of p-CHA and p-CHDA are unknown and therefore it is difficult to determine whether the underlying genes are regulated by *Rcc*. Biosynthesis of serotonin, on the other hand, is well characterized (Lee et al., 2008; Park et al., 2008; Back et al., 2016) in other species. We identified putative candidates in the barley genome and found these strongly up-regulated at 12 dpi confirming the metabolite analyses (Supplementary Table 1). Furthermore, several tyrosine/tryptophan decarboxylases, likely candidates for the TDC enzyme important for serotonin biosynthesis, are highly induced at early time points and belong to the highest induced DEGs in the analysis at 12 dpi. Together, this indicates that changes associated with *Rcc* infection at the metabolite level can be detected using our standard procedure, though to a limited resolution when compared to those at the transcriptome level.

## Discussion

Previous analyses of transcriptional responses of Fairytale infected by DK05 focused on the overall responses of barley to *R. collo-cygni* during pathogenic interaction (Sjökvist et al., 2019). Here we provide a comparative phenotypic, transcriptional and metabolite analysis of responses that includes Tipple, a cultivar tolerant to RLS, as well as responses to a milder *Rcc* isolate, the NZ11. Transcriptional responses of the two cultivars were ample, and our detailed analysis of their regulation at three time points after infection identified a large number of barley genes responding differently in the two cultivars, or in the presence of the two isolates (Figure 3). In contrast to the transcriptome responses, the metabolite analyses by HPLC identified only a limited number of compounds to be differentially accumulated in the infected leaves at the analyzed time points (Figure 7), indicating that a limited resolution of the response was provided by this analysis. Nonetheless, the levels of *p-*CHDA were found to increase significantly in both cultivars infected by DK05 from 3 dpi. *p-*CHDA is a derivative of *p*-CHA previously found to accumulate in the epidermis of powdery mildew-infected barley leaves (von Röpenack et al., 1998), and its increased level could be further investigated in a larger panel of cultivars as a possible early marker for pathogenic *Rcc* infection.

We found that RLS symptoms induced by the same isolate in the two cultivars are not correlated with the amount of fungal biomass, since Tipple leaves showed higher levels of *Rcc*. However, most likely the different responses induced by the two isolates might be a reflection of their differential growth capacity, since NZ11 showed only a threefold increase in biomass at 12 dpi compared to initial inoculum, while DK05 had a fivefold increase (Figure 1). Nonetheless, both isolates were identified in the apoplast with hyphae making direct contact to mesophyll cells that responded with cell wall thickening at connecting points (Figure 2). The role of the fungal ECM for *Rcc* colonization remains unknown, but it could be speculated they play a role in effector and/or phytotoxin delivery. Plant cell wall reinforcements are common responses of hosts to invading pathogens to decrease successful cell penetration and counteract further tissue colonization (Friend, 2016), and identifying such structures here, are confirmations for pathogenic responses in *Rcc*-barley interaction also at ultrastructural level. The interaction observed here is similar to that of the endophytic fungus *Epichloë festucae* in *Lolium perenne*, in its asymptomatic stage, was reported to have hyphae firmly attached to the host cell wall through an extracellular matrix (Tanaka et al., 2006; Christensen et al., 2008), resembling those reported here for *Rcc*. However, *L. perenne* did not induce either callose or lignin deposition during colonization by *E. festucae* (Rahnama et al., 2018). *Rcc* DNA has been detected in asymptomatic barley plants, and it will be interesting to know whether a similar phenotype, lacking the cell wall thickening, as observed in *E. festucae*- infected *L. perenne* is present during the endophytic lifestyle of *Rcc*. Cell wall modification in response to pathogen attack may involve callose (Meyer et al., 2009) or lignin deposition to prevent diffusion of toxins produced by the pathogen (Sattler and Funnell-Harris, 2013). *Rcc* produces rubellin, a photoactive anthraquinone toxin that induces peroxidation of membrane fatty acids and pigments oxidation resulting in leaf necrosis and chlorosis (Heiser et al., 2003). Future studies might determine whether the contact sites between *Rcc* and barley cells identified here represent also the sites where toxins are delivered, and as a consequence the host responds with cell wall thickening. Nonetheless, transcriptional responses of genes coding cell wall modifying enzymes have been identified especially in 12 dpi-infected samples (Supplementary Figure 5) likely reflecting the observed morphological changes in barley cell wall.

**FIGURE 8:**
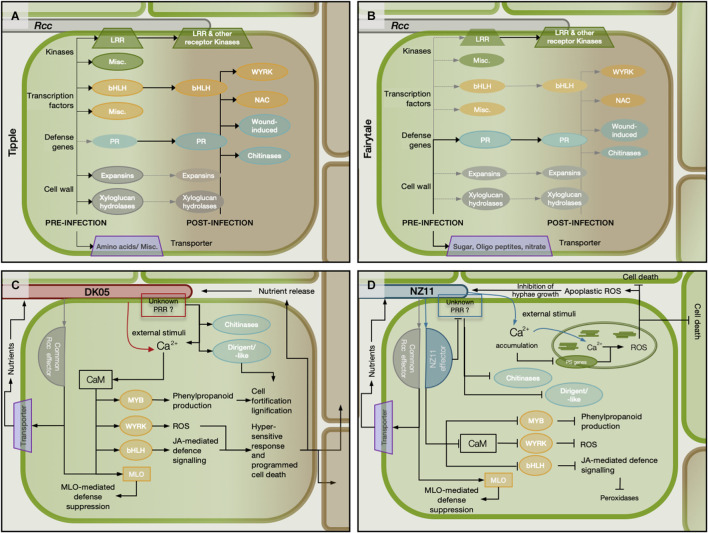
Schematic representation of potential modulation of the barley (*Hordeum vulgare L*) regulatory network during the infection with two different isolates of *Ramularia collo-cygni*. **(A,B)** Depict the expression of specific groups of genes before and after infection in the barley cvs Tipple and Fairytale, respectively. The focus is on expression differences between the two barley cultivars. **(C)** Schematic representation with the more aggressive isolate DK05. **(D)** Modulation of the host network during infection with the mild *Rcc* isolate NZ11. Solid lines with arrowheads represents higher expression before infection in the respective cultivar and activation of gene expression by *Rcc* colonization. Solid lines with blunt ends depict down-regulation of gene expression. Dotted gray lines with arrowhead show genes that are not or very low expressed.

Tipple and Fairytale vary greatly in their transcriptional and phenotypic responses to *Rcc* throughout the analyzed time points. Most differences are at the early (3 dpi) and late (12 dpi) time points indicating that the two cultivars have a differential capacity to recognize *Rcc* as a pathogen. This early differential response could be a result of their native expression level for genes important for pathogenicity, which we found to be numerous (Supplementary Figure 14). When compared to Fairytale, Tipple had an higher level of expression for more than 160 genes coding receptor kinases, transcription factors, defense-related proteins or transporters (Figures 8A,B), whose members may have a direct impact on cultivar responses, especially knowing that many of these are regulated by *Rcc* and similar members have been identified important for plant-pathogen interactions (Lemmens et al., 2005; Cook et al., 2015; Li et al., 2016). These genes could represent valuable marker genes for evaluating barley cultivars for improved tolerance to *Rcc*.

DK05 and NZ11 induce as well a differential response in the two cultivars with Fairytale showing the largest difference. Effector proteins are known to vary between fungal isolates and to contribute to host-dependent responses due to their interaction, or lack thereof, with cultivar-specific proteins (Lewis et al., 2012; Zabala et al., 2015; Zhou et al., 2015). It is likely that effectors differ between NZ11 and DK05 and these, by targeting specific host proteins, may contribute to the large differences in transcriptional responses presented here. We found it interesting that barley calmodulins (CaM) and associated genes were down-regulated at 3 dpi and only marginally regulated at 7 dpi in the NZ11*-*Fairytale interaction. Moreover, CaM and Calcium-signaling associated genes were identified as hub-genes in 4 modules (*orange*, *purple*, *cyan*, *magenta*), supporting the involvement of CaM and Ca^2+^-binding proteins in this interaction. CaM has been shown to interact with transcription factors from bHLH, WRKY, NAC, or MYB family (Park et al., 2005; Galon et al., 2010; Zeng et al., 2015), and we found as well that more WRKY and MYB TFs were either not or down-regulated in the Fairytale-NZ11 interaction. Interestingly, this expression pattern coincided with the down-regulation of genes in the core phenylpropanoid pathways (CPP), where two phenylalanine ammonia-lyases (PAL) and one cinnamate 4-hydroxylase (C4H) were found down-regulated in Fairytale at 3 dpi during NZ11 infection. By contrast, the same genes were up-regulated or not significantly regulated during infection by DK05, nor in Tipple samples.

The *Mlo* gene has been previously suggested to have contributed to the emergence of RLS in barley (McGrann et al., 2014). Cultivars analyzed here have the wild-type *Mlo* gene variant, and we found a marginal increase in transcript levels for *Mlo* and *Mlo*-like proteins at 3 dpi in Fairytale during infection by DK05 interaction has been reported (Sjökvist et al., 2019). We identified variation between cultivars for timing and direction of regulation of *Mlo* and *Mlo*-like genes regulation. Tipple infected by DK05 showed a significant increase in *Mlo* transcript already at 3 dpi, while Fairytale only at 7 dpi. Furthermore, the *Mlo*-like protein 1 (HORVU0Hr1G008830) was identified as a hub gene in the *lightgreen* module containing genes regulated in response to *Rcc* inoculation (Figure 5B). Together this indicates that MLO proteins are contributors to barley responses to pathogenic *Rcc* (Figure 8).

Importantly, the regulation of defense genes was primarily driven by the cultivar, rather than the isolate (Supplementary Figure 8). Nonetheless we found genes coding chitinases and dirigent-like proteins previously described as early plant defense responses to be differently regulated by DK05 and NZ11 at 3 dpi. Dirigent and dirigent-like proteins participate in lignin and lignan formation by promoting monolignol coupling (Guo et al., 2012). The accumulation of lignans is connected to the basal plant defense, but they are also known to act as phytoalexin with anti-microbial activities (Burlat et al., 2001). The up-regulation of dirigent-like proteins during DK05 colonization indicates an early recognition of this isolate as a putative pathogen and possibly earlier onset of cell-wall fortification as observed in the electron micrographs (Figure 2).

Genes involved in photosynthesis were also found to be differentially regulated by the colonization of the two isolates (Supplementary Figure 6). NZ11 infection tends to cause 5 – 6 times more regulated genes than DK05. Infections of *Pseudomonas syringae* on Arabidopsis have shown an early down-regulation of photosynthesis transcripts after infection, leading to ROS burst in chloroplasts and the apoplast suppressing *P. syringae* colonization without the initiation of programmed cell death in the host (Gohre, 2015; Zabala et al., 2015). In the context of our study, the stronger down-regulation of photosynthetic genes during NZ11 infection indicates the possibility of an isolate-specific signaling leading to reactive oxygen species (ROS) production without evoking programmed cell death. ROS released from the apoplast might contribute to the slower colonization observed and hence the lower fungal biomass we reported for NZ11 (Figure 1).

Together, our comprehensive data from transcriptional responses combined with metabolite and phenotypic analyses provide a solid basis for identification of candidate marker genes and metabolites that can be further monitored and explored in high throughput analyses of barley cultivars for improved tolerance to *Rcc*. Further research aimed at characterizing fungal responses and identification of isolate specific effectors will shed light on the molecular cross-talk between this pathogen and its host.

## Data Availability Statement

The original contributions presented in the study are publicly available. This data can be found here: National Center for Biotechnology Information (NCBI) BioProject database under accession number PRJEB14791.

## Author Contributions

RL designed wet-lab experiments and dry-lab analysis, prepared fungal cultures and plants, performed fungal inoculation on host plants, subsequent sampling of plant material, RNA, DNA, metabolite extractions, RNA quality controls, qPCR for fungal DNA measurement, performed the final differential expression (DE) analysis, performed the WGCNA, network analysis, gene ontology (GO) analysis as well as metabolite analysis and identification, interpreted RNA-seq data, prepared figures and tables, and wrote and edited the manuscript. ES designed dry-lab analysis, performed RNA-seq data quality control, contributed to read mapping, quality control, read counting, normalization (TPM values), designed and performed the initial differential gene expression analysis, provided guidance for further analyses, contributed to the WGCNA and GO analysis, prepared Supplementary Figure 1, and commented on the manuscript. All authors contributed to the article and approved the submitted version.

## Conflict of Interest

RH is employed by Sejet Plant Breeding. The remaining authors declare that the research was conducted in the absence of any commercial or financial relationships that could be construed as a potential conflict of interest.

## Publisher’s Note

All claims expressed in this article are solely those of the authors and do not necessarily represent those of their affiliated organizations, or those of the publisher, the editors and the reviewers. Any product that may be evaluated in this article, or claim that may be made by its manufacturer, is not guaranteed or endorsed by the publisher.
