# Supplementary material for: Deciphering Molecular Host-Pathogen Interactions During Ramularia Collo-Cygni Infection on Barley
Source: Front Plant Sci. 2021 Oct 22;12:747661. doi: 10.3389/fpls.2021.747661 (PMC8570322; doi:10.3389/fpls.2021.747661)
Supplement: Supplementary file 2 [file Data_Sheet_2.docx]

Material and Methods - Supplement

*Plant and fungal growth conditions*

Barley (*Hordeum vulgare*) cvs. Fairytale and NFC Tipple (Tipple, hereafter) were cultivated at 19 °C in 16 h light, 300 μmol m^–2^s^–1^ and 8 h darkness. Plants with fully developed 2^nd^ leaves (14 days old) were used for downstream experiments. *Ramularia collo-cygni* (*Rcc*) isolates DK05 and NZ11 were grown in darkness on PDA plates supplemented with Streptomycin (10 µl/ml). Rcc liquid cultures was prepared 3 weeks before inoculation by transfer of 3 agar plugs into 75 mL PDB media supplemented with Streptomycin (10 µl/ml). Cultures were grown at room temperature in the dark and under constant agitation at 120 rpm until used.

*Fungal inoculation*

Leaves were fixed horizontally on a plastic support with cotton strings facing the adaxial side upwards. Fungal hyphae were filtered using a cotton cloth, subsequently washed with ddH_2_O (500 ml) and diluted in 15 ml ddH_2_O. Sonication was used to break down the hyphae in smaller pieces (3 times 30 s). Before collection of the supernatant, the hyphal solutions were rested for two minutes to allow larger hyphae to settle. Hyphal concentration was assessed using a Fuchs-Rosenthal counting chamber and adjusted to 5x10^5^ hyphae/ml with Tween 20 solution (4 µl/ml). The suspension was used to spray inoculate with an atomizer (DESAGA, Sarstedt-Gruppe; NS18,8/26) until run off. Plants were kept for 48 h in the dark under plastic covers to maintain high relative humidity (80–100%). Subsequently, plants were grown at a normal 16h/8h light-dark cycle. Control plants were mock-inoculated with water and Tween 20 (4 µl/ml) and maintained in the same conditions as the inoculated plants. Symptom development on leaves was monitored on a daily basis, and samples were collected at 3, 7, 12 days post infection (dpi). Leaf segments from 3 different leaves were pooled. Biological replicates were derived using independently grown cultures of Rcc inoculum.

*DNA and RNA extractions*

We used a modified protocol from Yang *et al*. (2008) do extract DNA and RNA from the same sample and described in our previous publication (Sjökvist *et al*., 2018). In brief, snap-frozen samples were broken down to a powder using a tissue-lyser, re-suspended in 900 µl of Extraction buffer (2% CTAB, 0.1 M Tris-HCL (pH 8), 1.4 M NaCl (5 M), 20 mM EDTA (pH 8), 2% PVPP) and 100 µl β-mercaptoethanol and kept for 10 min at 65 °C. Chloroform (1 mL) was added to the samples, well mixed to form emulsion and centrifuged for 20 min at 4 °C at 10000 g. The supernatant was transferred to a new tube and the same volume of phenol:chloroform:isoamylalcohol (25:24:1) was added. Centrifugation was performed as before. The top aqueous layers were transferred to a new tube and mixed with 1 volume of chloroform:isoamylalcohol (24:1) and centrifuged as before. LiCl (8 M) (a third of the volume) was added to the supernatant and incubated overnight at -20 °C. The next day, samples were centrifuged for 30 min at 4 °C with 10000 g, the pellet washed twice with 70% EtOH and then air dried. To the supernatant of the RNA precipitation was added 2 volumes of 96% EtOH and 1/10 volume of NaCl (5 M), incubation for 20 min at 20 °C followed by centrifugation for 30 min. The DNA pellet was washed with 70% EtOH. DNA and RNA were dissolved in 50 µl ddH_2_O and stored at -20 °C.

*Fungal biomass measurement*

*R. collo-cygni*primers were designed for the internal transcribed spacer (ITS) region (GenBank KJ504786.1) and amplified a 174-bp fragment (ITS forward primer 5′-TCC AAC CCT TTG TGA ACG CAT-3′ and the reverse primer 5′-ATT TCG CTG CGT TCT TCA TCG A-3′). We used the elongation factor gene (EFO) from barley, to assess the relative abundance of fungal RNA to plant DNA (EFO forward primer 5′-ACC CTG ACA AGG TTC CCT TC-3′ and the reverse primer 5′-ACC AGT CAA GGT TGG TGG AC-3′). The quantitative PCR was performed with the BioRad CFX384 real-time PCR detection system, using the KAPA SYBR Fast qPCR mix.

*RNA-seq analysis of infected and uninfected samples*

Software versions, parameters and references were used as published before by Skökvist and coworkers (Sjökvist *et al*., 2018). RNA samples were sent to Edinburgh Genomics, where they RNA libraries (TruSeq stranded mRNA-seq kit) and sequencing (Illumina HiSeq4000, 150 bases paired end) were carried out. Samples were split over six lanes. FastQC report for fastq filels were summarized using MultiQC. We trimmed adapters and removed low-quality base calls using Skewer. Raw data have been stored in the ENA read archive under BioProject PRJEB14791.

*RNAseq read mapping and read counting*

In brief, in order to separate RNA reads from host and fungus, we performed competitive mapping. We merged the barley (Mascher *et al.*, 2017) and Rcc genome (McGrann *et al*., 2016) to one file using samtools (Li *et al.*, 2009). The high confidence gene prediction for barley and our Rcc predictions were merged into one gtf file. We used STAR alignment with the 2-pass approach in order to map the trimmed read (Dobin *et al*., 2013). Quality of mapping and sequencing depth was checked with RSeQC and MultiQC (Ewels *et al.*, 2016, Wang *et al.*, 2012). We counted the RNA reads using HT-seq by performing a per-gene base counting where a read pair overlaps an exon (Anders *et al*., 2015) and subsequently counts were split based on species. In this study we only used the barley counts for further analysis.

*TPM calculation*

TPM (Transcripts per million) values were calculated using RSEM (Li and Dewey, 2011). For an accurarate calculation of fractions per library for each species we split the reads before re-mapping and counting. Bam files from competitive mapping were also split. First the split was made on contig, then merged based on sample and species. Next reads, which were properly paired, were extracted from each bam file using Samtools (Li *et al.*, 2009). We mapped the reads using Star (Dobin *et al*., 2013), after which RSEM was run on bam files. A Trinity script was used to normalize the TPM values using the Trimmed Mean Method (TMM) and format the output (Haas *et al.*, 2013).

*Differential gene expression and GO enrichment analysis*

The *EdgeR* package (McCarthy *et al.*, 2012, Robinson *et al.*, 2010) was used to carry out the differential expression analyses on the HT-seq counts according to the GLM method. Our three biological replicates were taken into account in this approach. Comparing infected vs. control samples genes, those considered differentially expressed (log2 FC > 1, false discovery rate < 0.05) were filtered based on their TMM values, where only genes with a TMM value of 10 in at least 3 samples were included in further analyses. We identified barley genes expressed in the control samples of one or other barley variety (false discovery rate < 0.05). Genes with a logFC <= -2 were considered highly expressed in Tipple, whereas logFC >= 2 was considered as highly expressed in Fairytale.

GO-enrichment analyses were performed on the differentially expressed genes using GOtools. A generic slim file was downloaded from geneontology.org and used for creating a GO-association file from the barley GO-annotations with a consistent hierarchy and resolution. This file was used for GO-enrichment analyses together with each list of up- or down-regulated differentially expressed genes.

*Supplementary Figures*


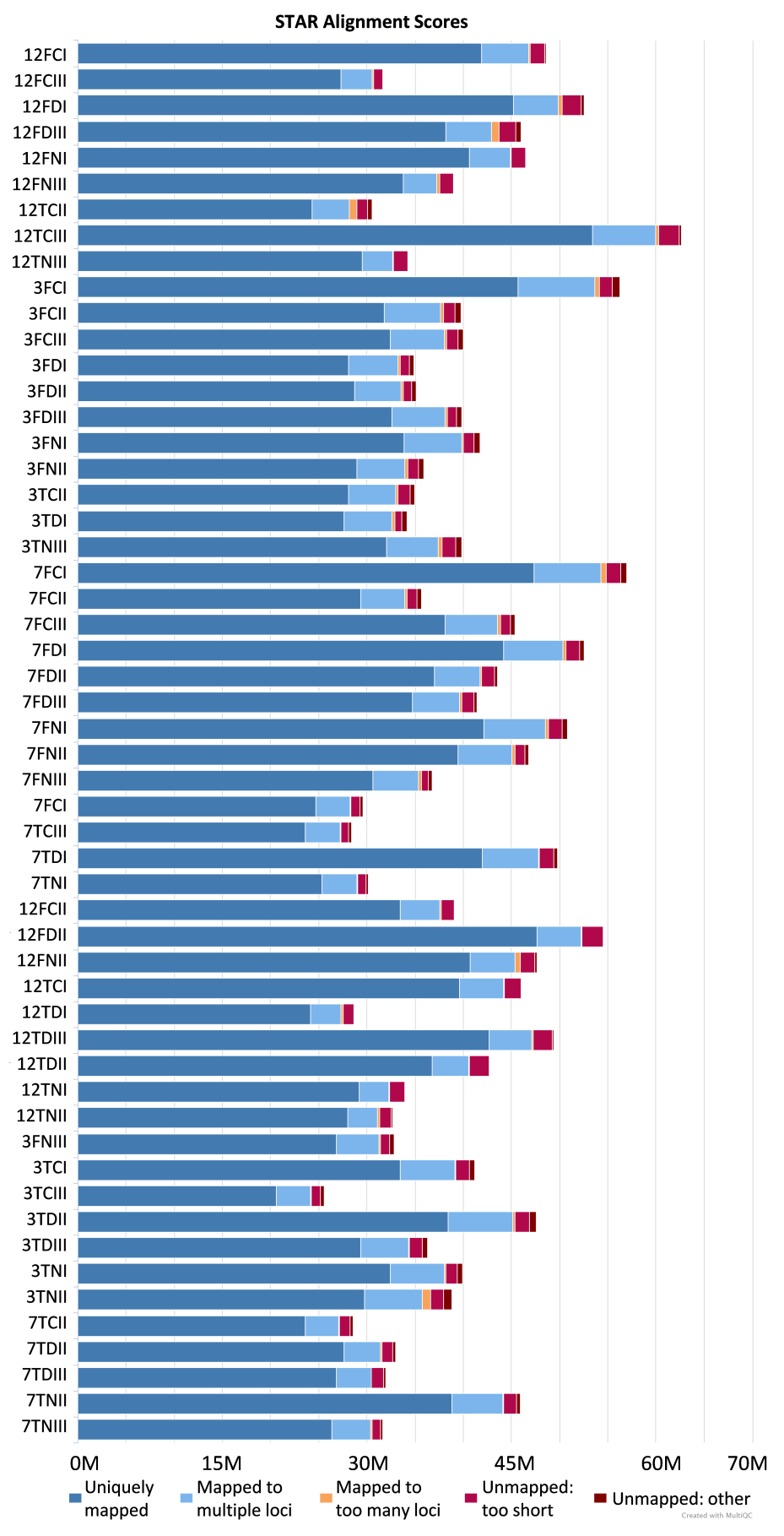


Supplementary Figure 1 Mapping quality across samples of the STAR alignment. Abbreviations: F – cv. Fairytale, C - control (un-inoculated), D – Rcc DK05 inoculated, 3, 7, 12- days after inoculation, 1, 2, 3 - biological replica.

LACK OF CONSISTENCY OF “cv” or “cv.” HERE TOO BETWEEN CAPTIONS AS WELL AS Rcc ITALICS


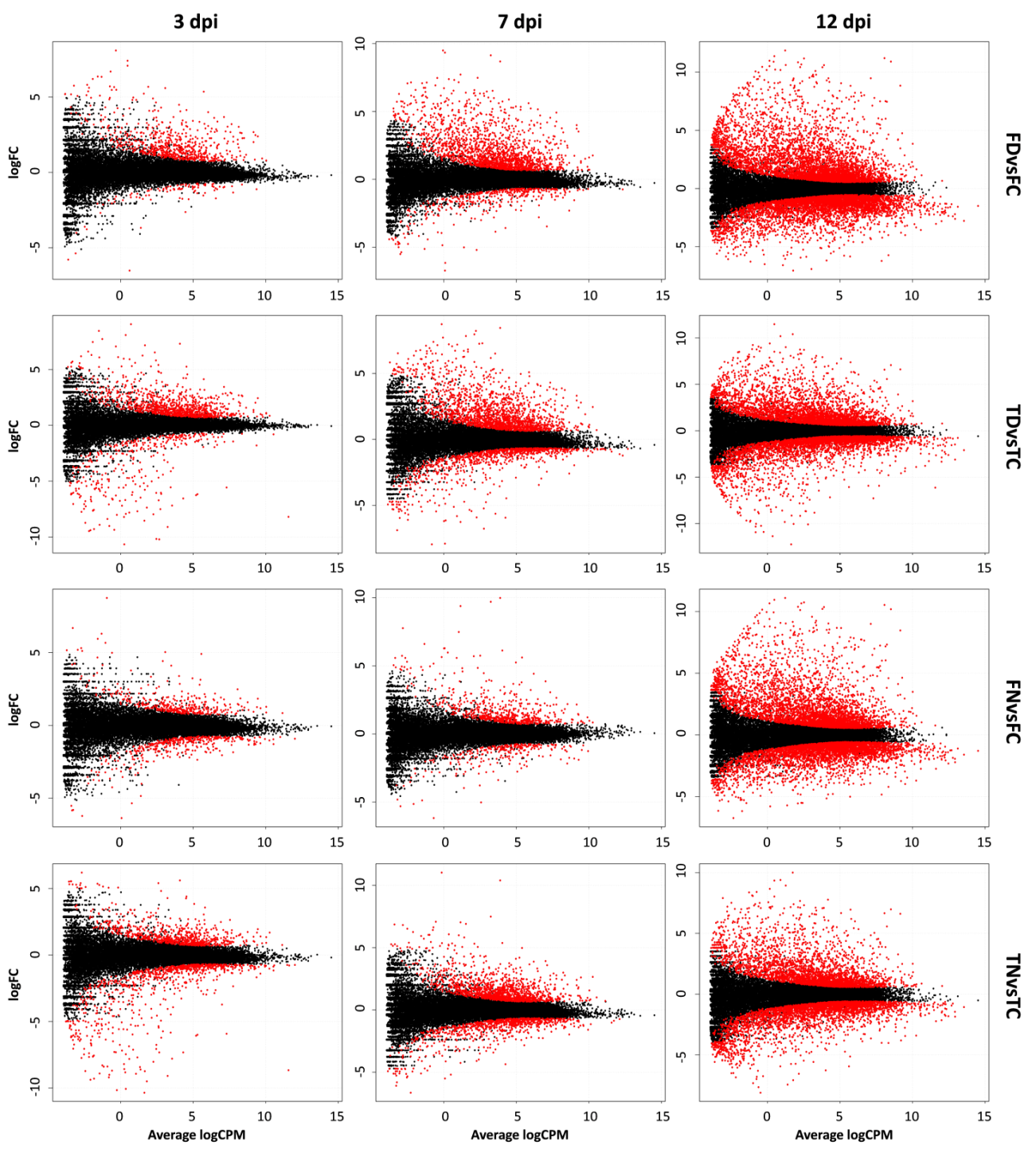


Supplementary Figure 2 Differentially expressed barley genes during Ramularia leaf spot disease progression. The distribution of differentially expressed genes (red) and non-regulated genes (black) when comparing non-infected barley leaves at the analyzed time points is shown. Abbreviations: F – cv Fairytale; T – cv Tipple, D – Rcc DK05, N – Rcc NZ11, logFC – log fold change, logCPM – log counts per million.


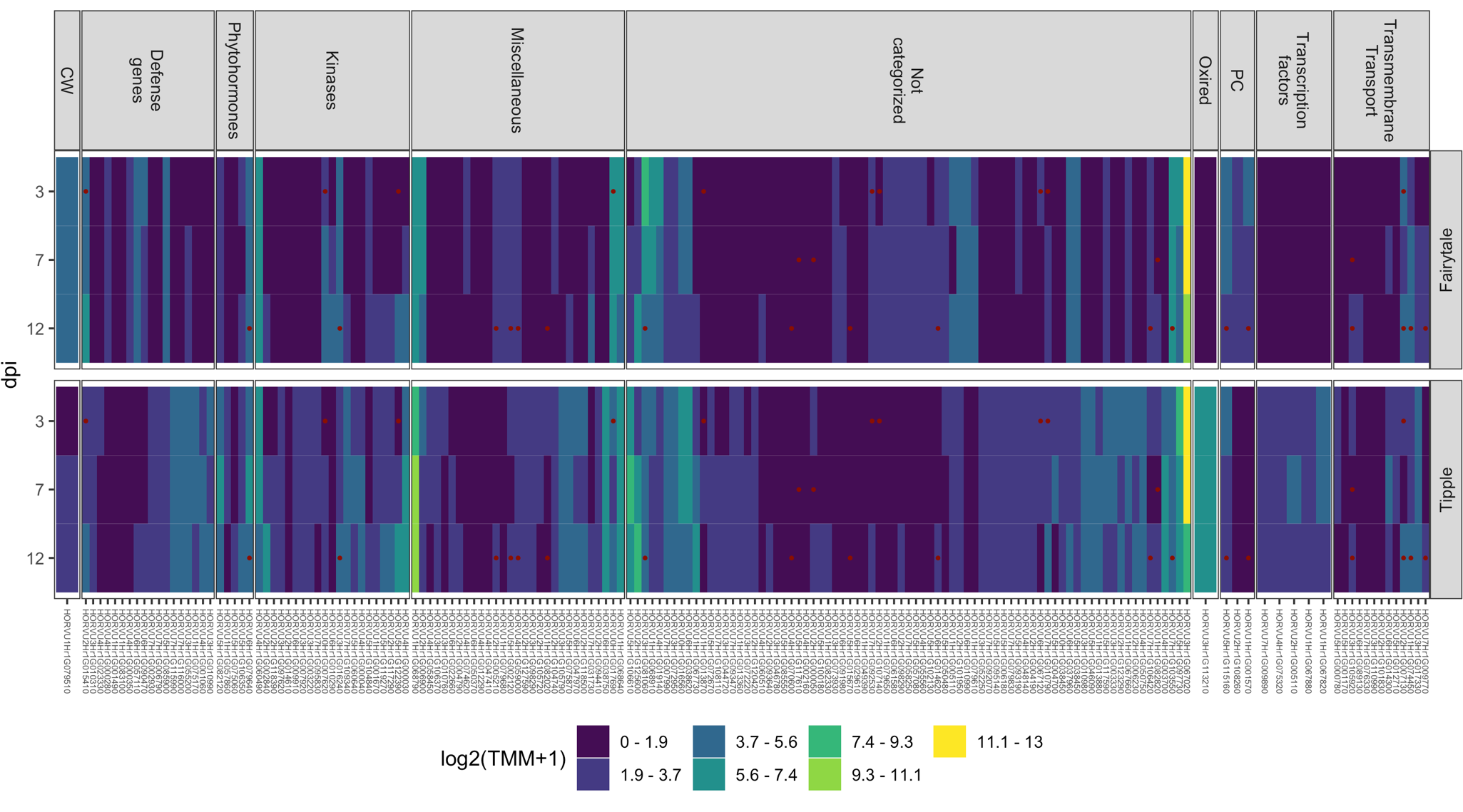


Supplementary Figure 3 Low differentially expressed barley genes between Tipple and Fairytale control samples. Barley genes that are significant differentially expressed between Tipple and Fairytale control samples and either not at all or low-regulated (FDR < 0.05, logFC -2 – 2) during Rcc colonization. The red dot indicates that the expression between the cultivar control samples were not significantly different at this time point. Abbreviations: TMM – Trimmed mean of M-values.


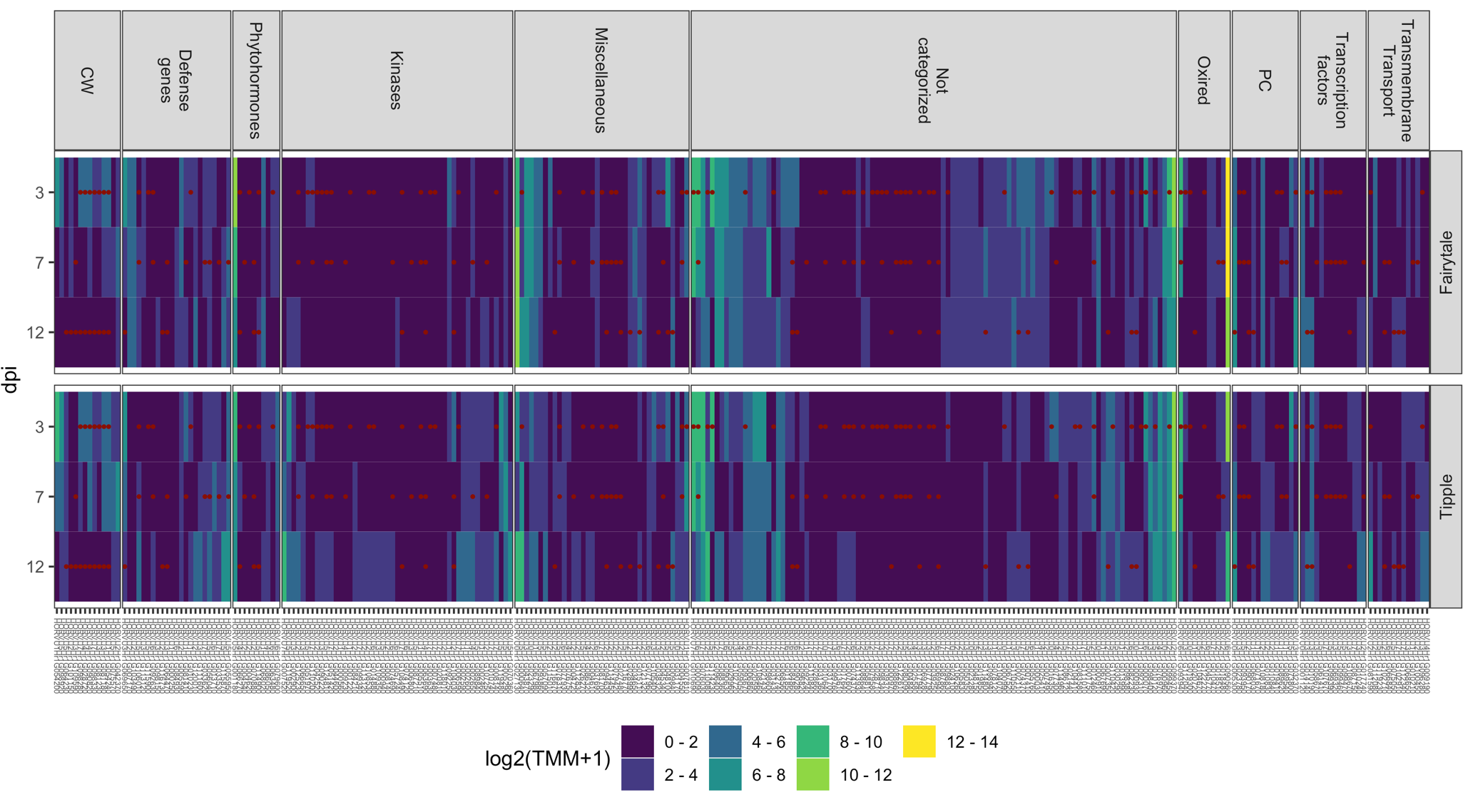


Supplementary Figure 4 Highly differentially expressed barley genes between Tipple and Fairytale control samples. Barley genes that are significant differentially expressed between Tipple and Fairytale control samples and also highly regulated (FDR < 0.05, logFC < -2 or logFC > 2) during Rcc colonization. The red dot indicates that the expression between the cultivar control samples were not significantly different at this time point. Abbreviations: TMM – Trimmed mean of M-values.


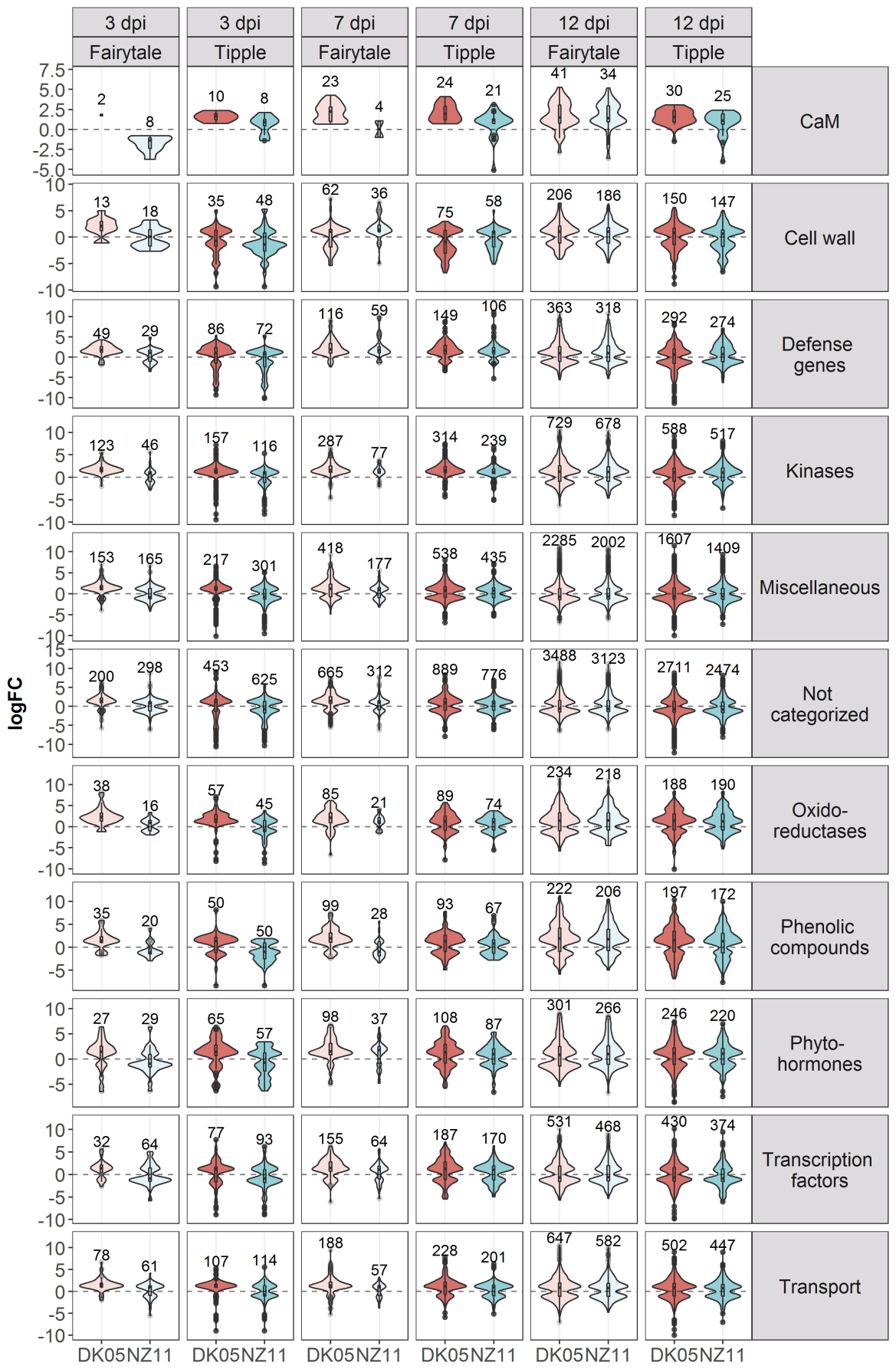


Supplementary Figure 5 Differentially expressed (DE) barley genes during Ramularia leaf spot (RLS) disease progression subdivided into MAIN MOLECULAR function. Violin plots with included box-whisker plots depicting the general trend of up and down regulation during RLS progression on barley (Hordeum vulgare L) cv. Fairytale and cv. Tipple during foliar infection with Rcc isolates DK05 and NZ11.


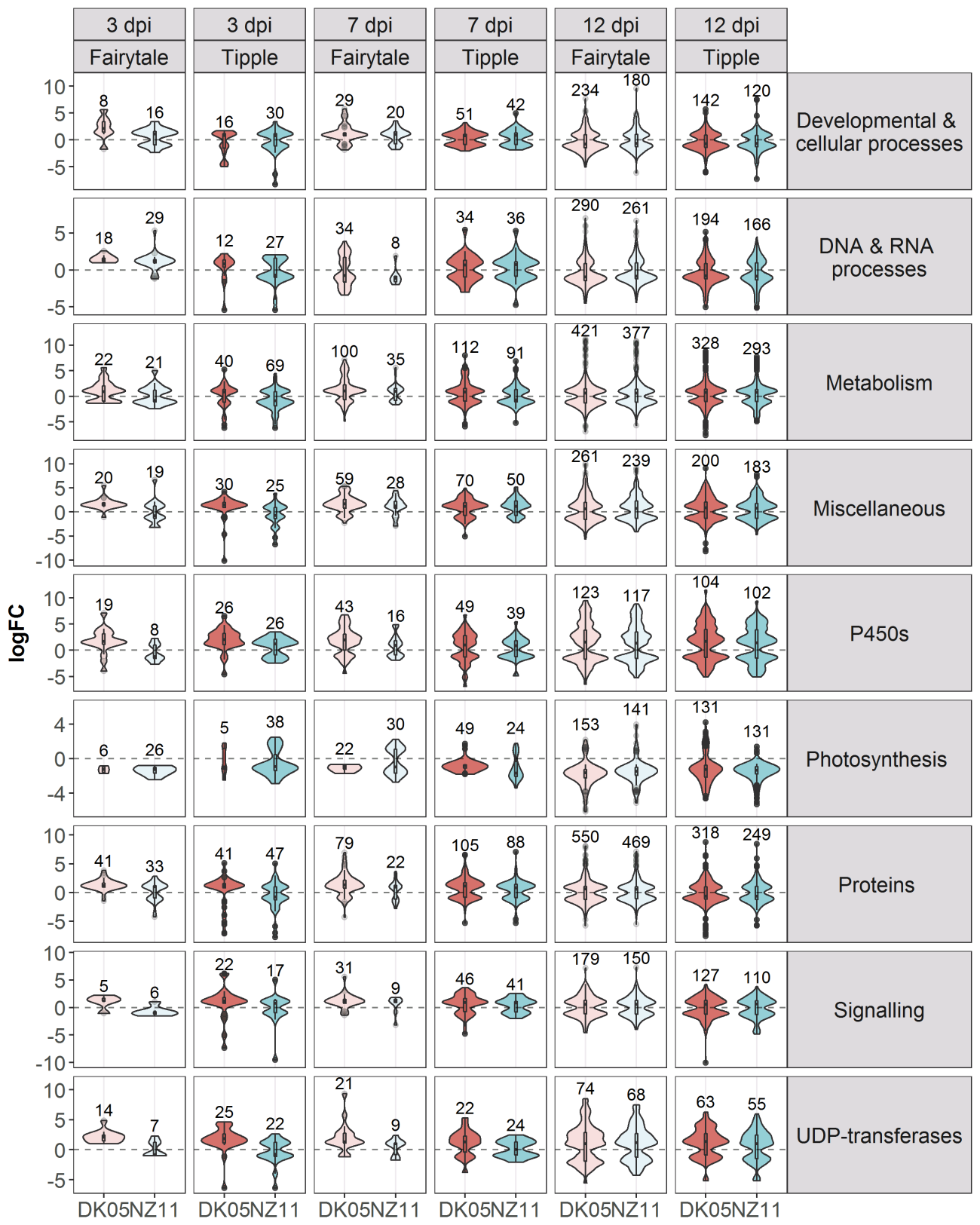


Supplementary Figure 6 Differentially expressed (DE) barley genes during Ramularia leaf spot (RLS) disease progression with different biological functions (MISCELLANEOUS group). Violin plots with included box-whisker plots depicting the general trend of up and down regulation during RLS progression on barley (Hordeum vulgare L) cv. Fairytale and cv. Tipple during foliar infection with Rcc isolates DK05 and NZ11.


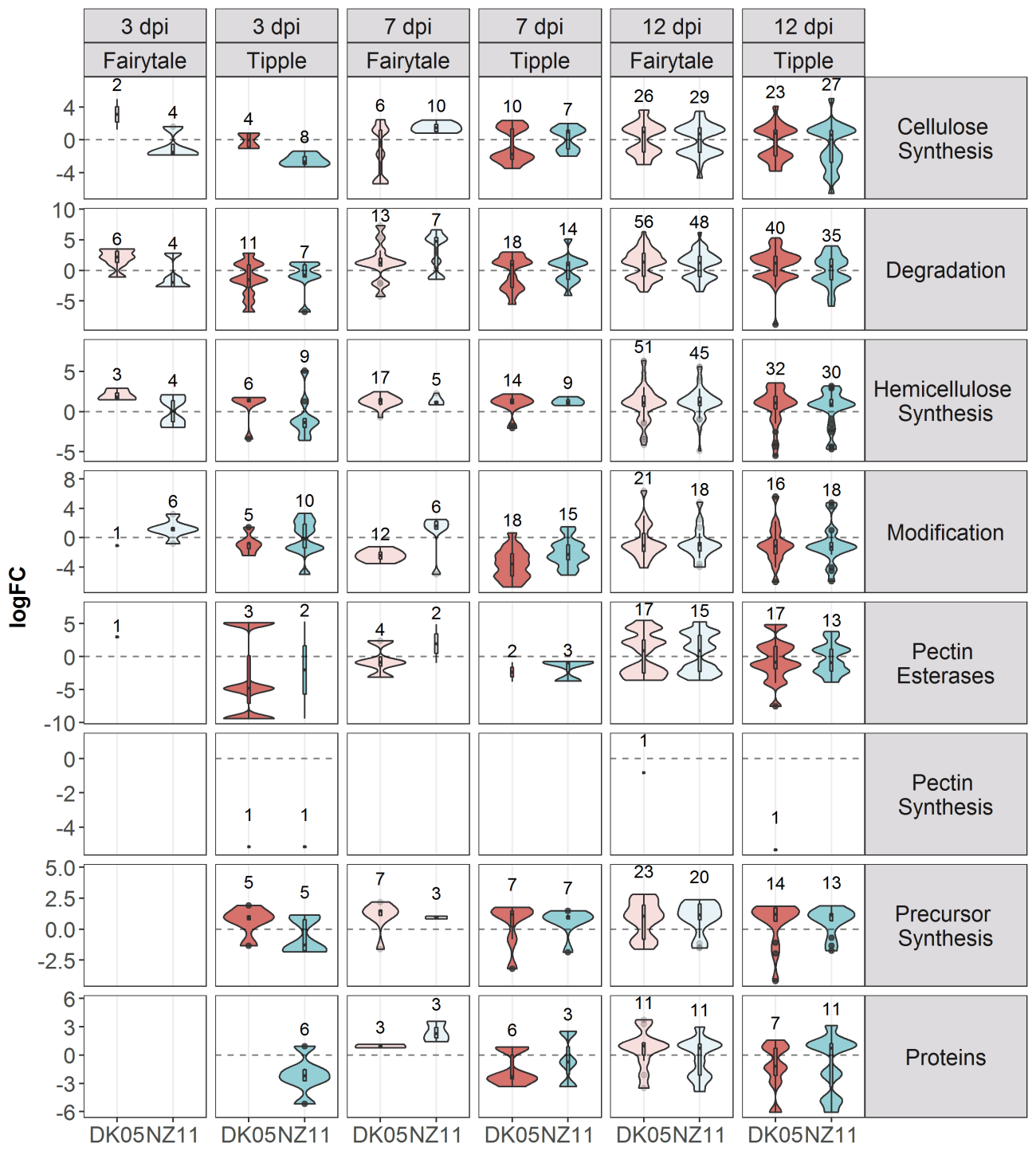


Supplementary Figure 7 Differentially expressed (DE) barley genes across different sub-categories of CELL WALL processes during Ramularia leaf spot (RLS) disease progression. Violin plots with included box-whisker plots depicting the general trend of up and down regulation during RLS progression on barley (Hordeum vulgare L) cv. Fairytale and cv. Tipple during foliar infection with Rcc isolates DK05 and NZ11.


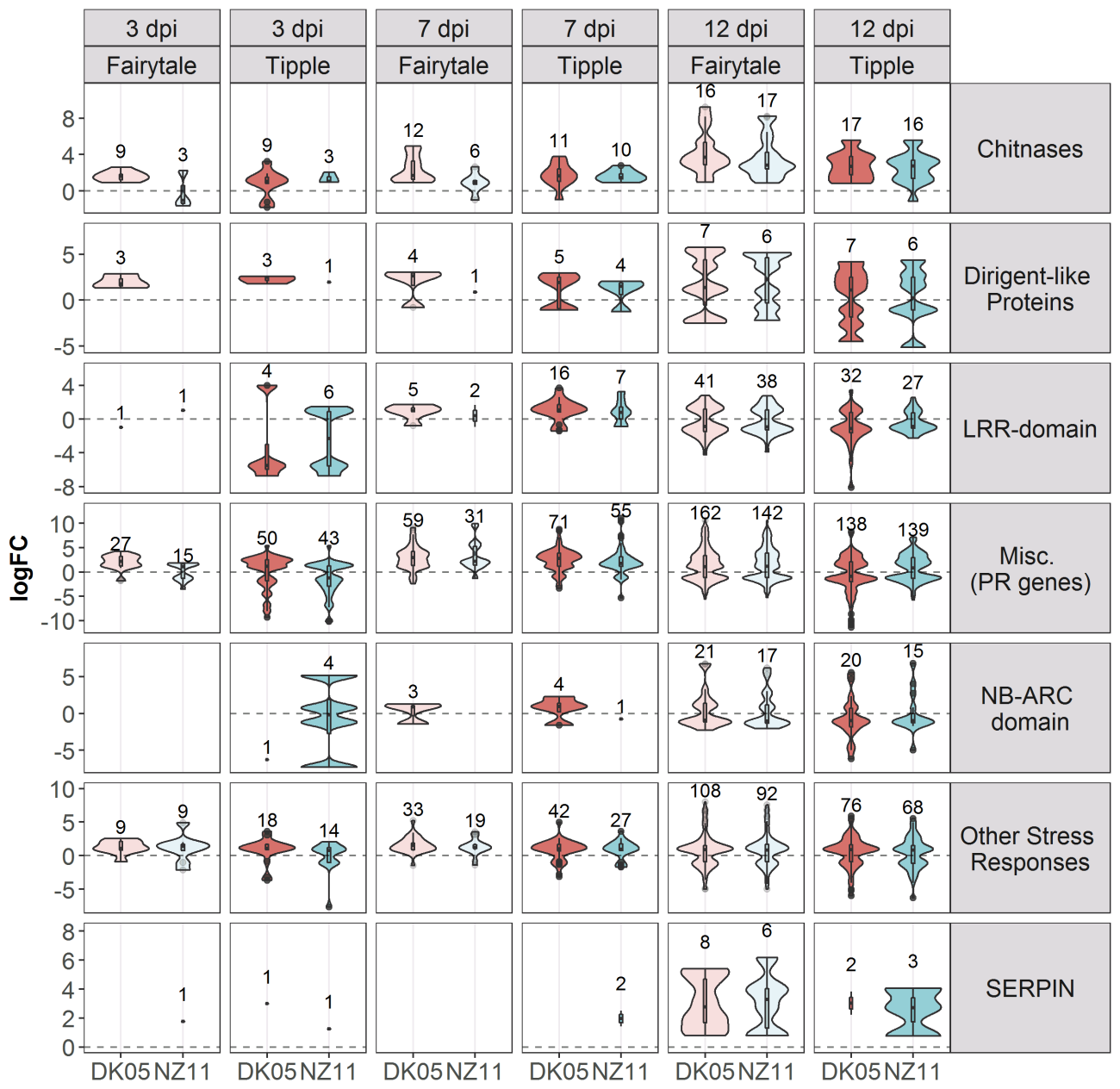


Supplementary Figure 8 Differentially expressed (DE) barley genes across different sub-categories of DEFENSE GENES during Ramularia leaf spot (RLS) disease progression. Violin plots with included box-whisker plots depicting the general trend of up and down regulation during RLS progression on barley (Hordeum vulgare L) cv. Fairytale and cv. Tipple during foliar infection with Rcc isolates DK05 and NZ11.


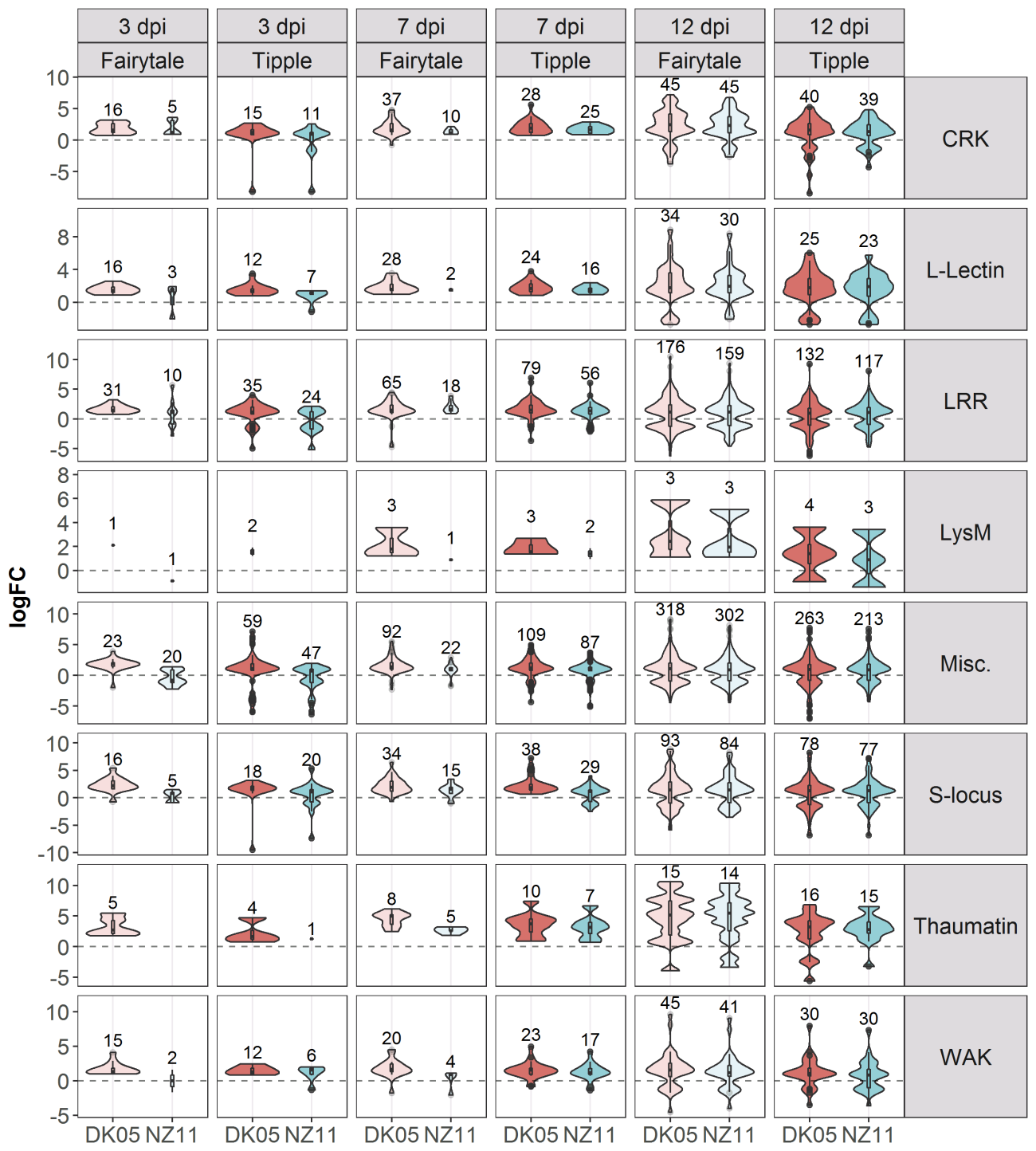


Supplementary Figure 9 Differentially expressed (DE) barley genes across different sub-categories of KINASES during Ramularia leaf spot (RLS) disease progression. Violin plots with included box-whisker plots depicting the general trend of up and down regulation during RLS progression on barley (Hordeum vulgare L) cv. Fairytale and cv. Tipple during foliar infection with Rcc isolates DK05 and NZ11.


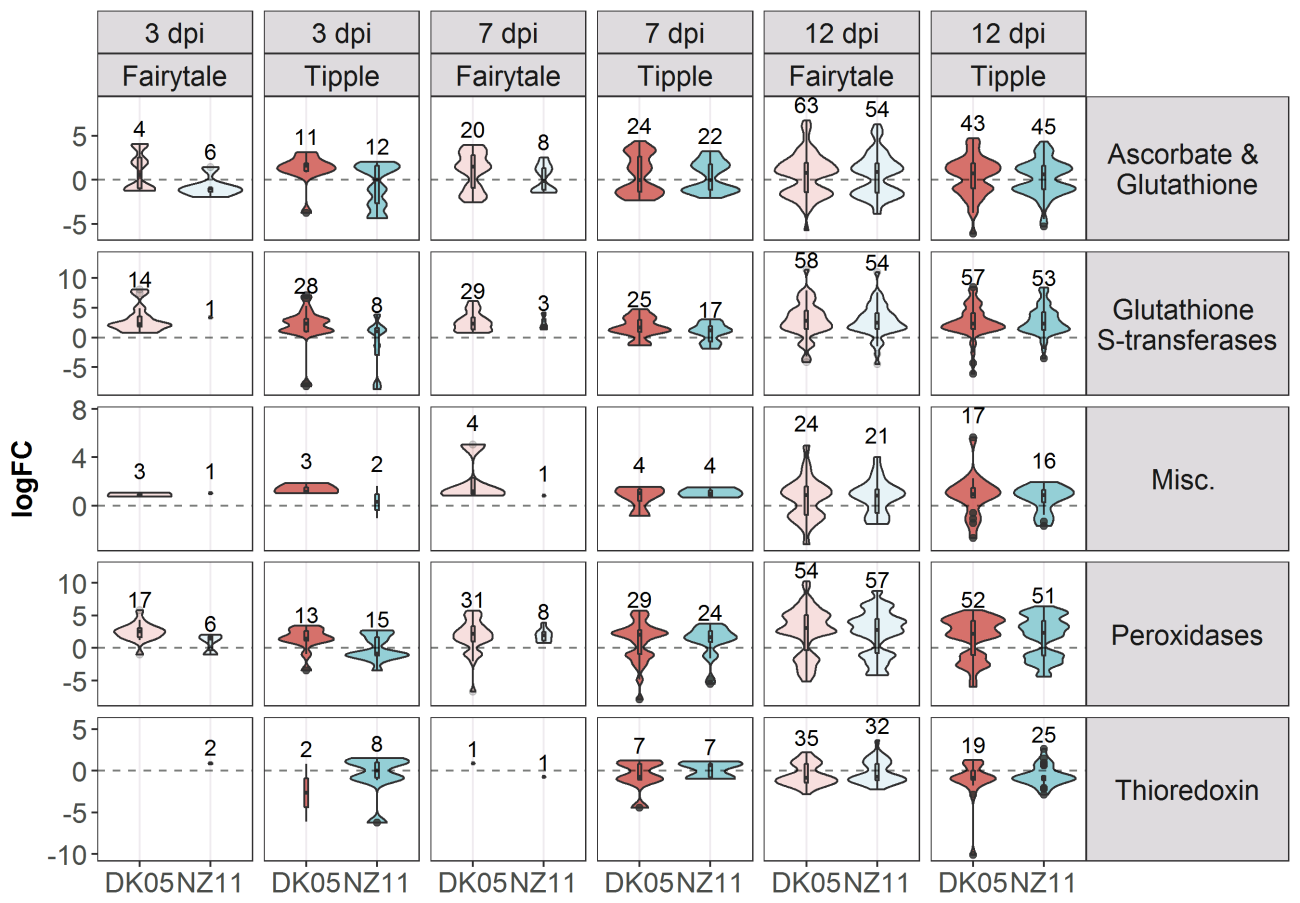


Supplementary Figure 10 Differentially expressed (DE) barley genes across different sub-categories of OXIDIOREDUCTASE during Ramularia leaf spot (RLS) disease progression. Violin plots with included box-whisker plots depicting the general trend of up and down regulation during RLS progression on barley (Hordeum vulgare L) cv. Fairytale and cv. Tipple during foliar infection with Rcc isolates DK05 and NZ11.


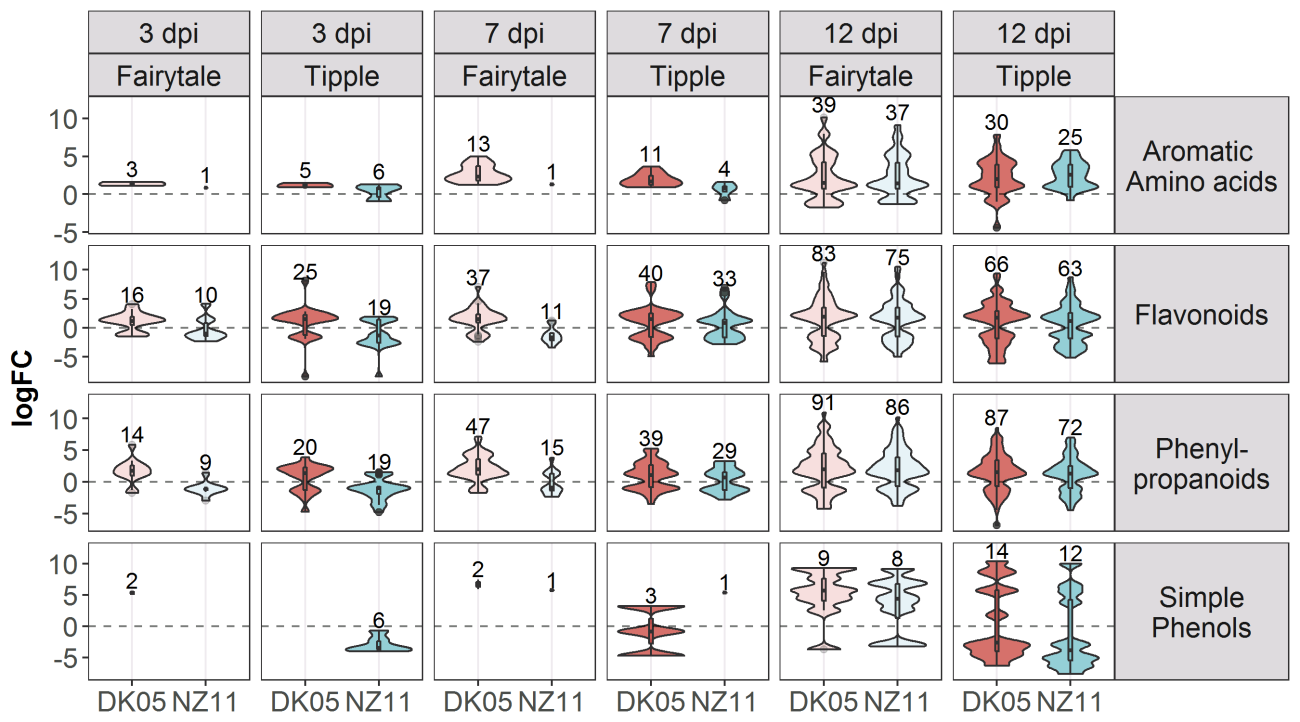


Supplementary Figure 11 Differentially expressed (DE) barley genes across different sub-categories of PHENOLIC COMPOUND biosynthesis during Ramularia leaf spot (RLS) disease progression. Violin plots with included box-whisker plots depicting the general trend of up and down regulation during RLS progression on barley (Hordeum vulgare L) cv. Fairytale and cv. Tipple during foliar infection with Rcc isolates DK05 and NZ11.


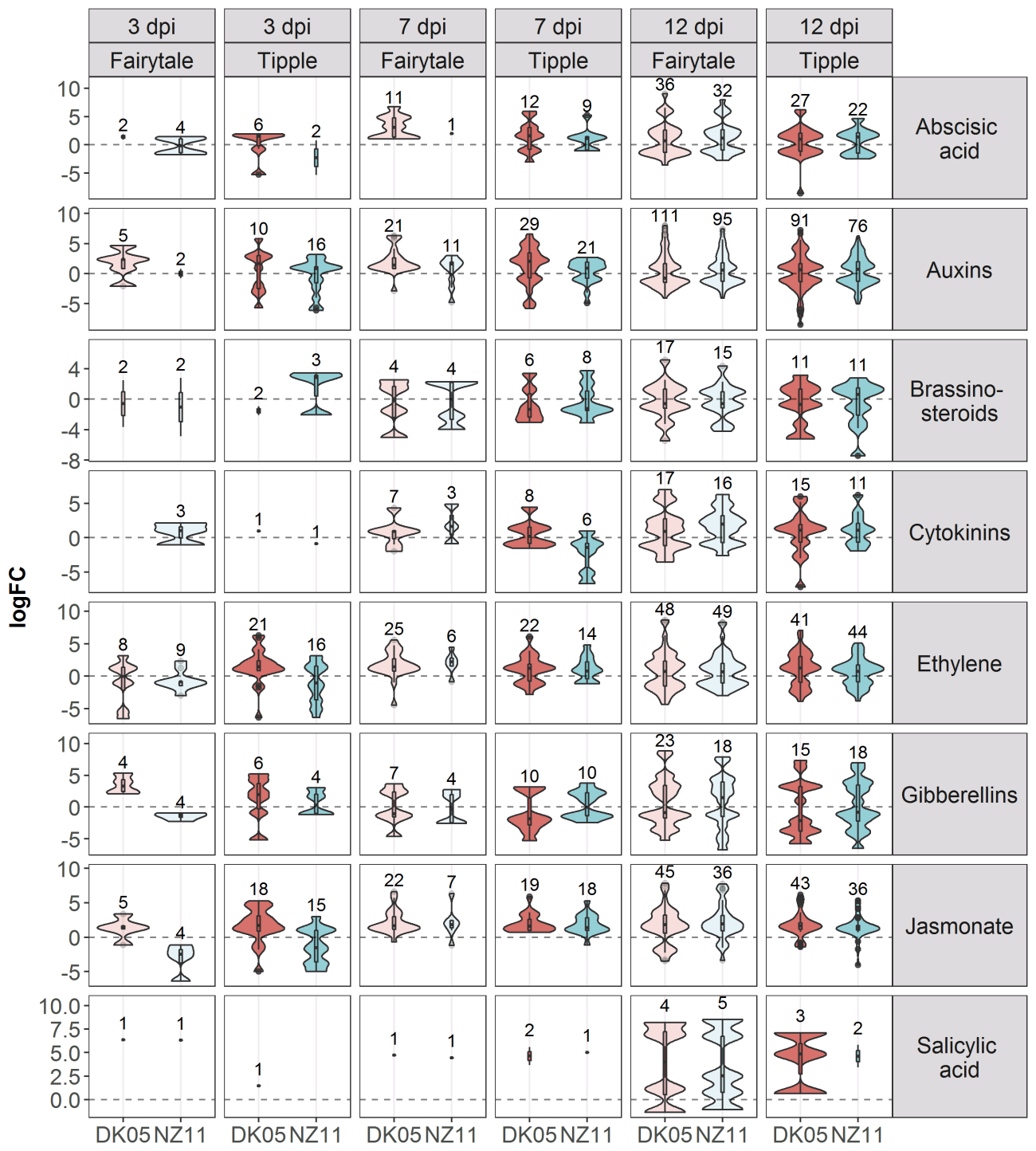


Supplementary Figure 12 Differentially expressed (DE) barley genes across different sub-categories of genes involved in PHYTOHORMONE biosynthesis and signaling during Ramularia leaf spot (RLS) disease progression. Violin plots with included box-whisker plots depicting the general trend of up and down regulation during RLS progression on barley (Hordeum vulgare L) cv. Fairytale and cv. Tipple during foliar infection with Rcc isolates DK05 and NZ11.


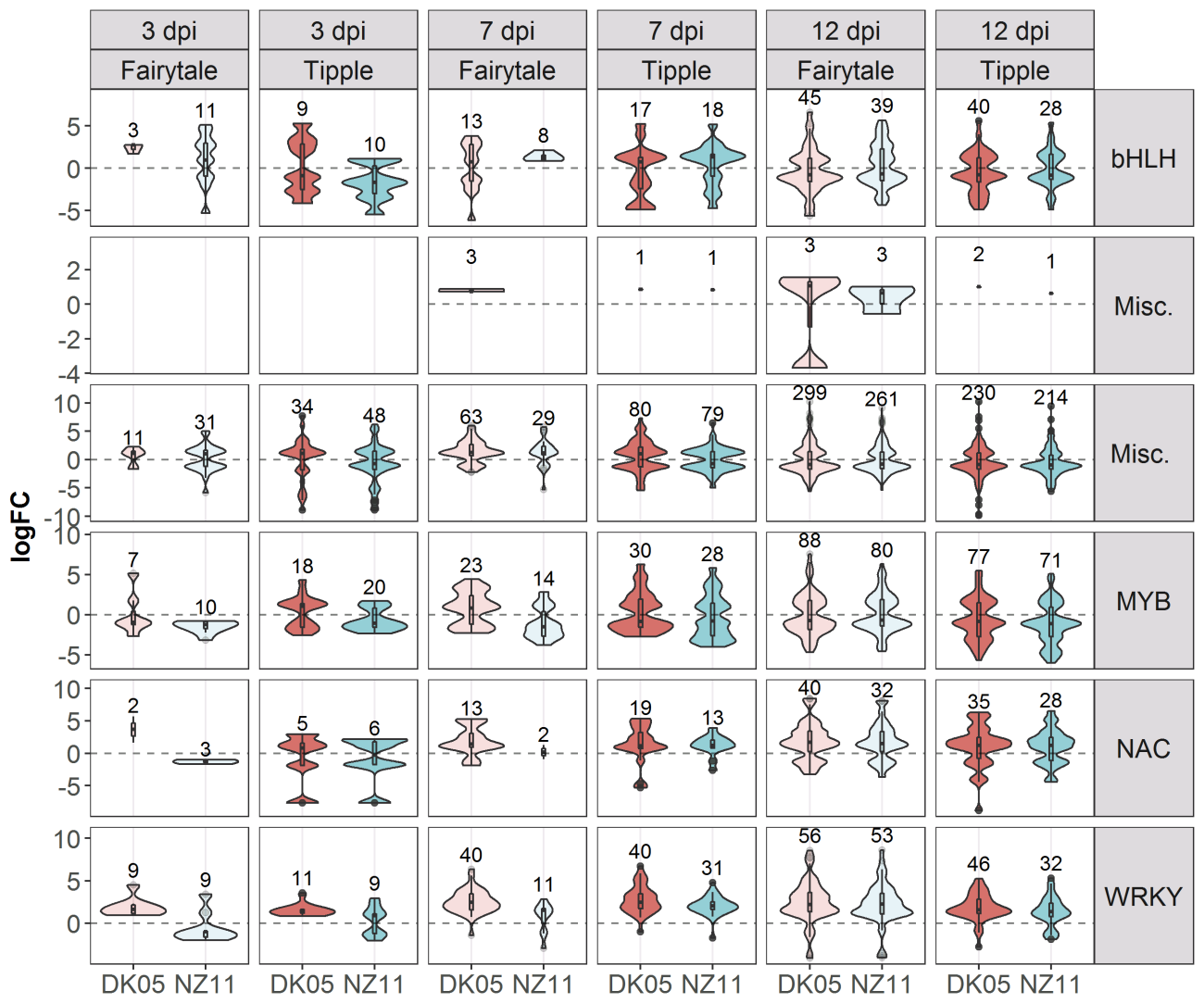


Supplementary Figure 13 Differentially expressed (DE) barley genes across different sub-categories of TRANSCRIPTION FACTORS during Ramularia leaf spot (RLS) disease progression. Violin plots with included box-whisker plots depicting the general trend of up and down regulation during RLS progression on barley (Hordeum vulgare L) cv. Fairytale and cv. Tipple during foliar infection with Rcc isolates DK05 and NZ11.


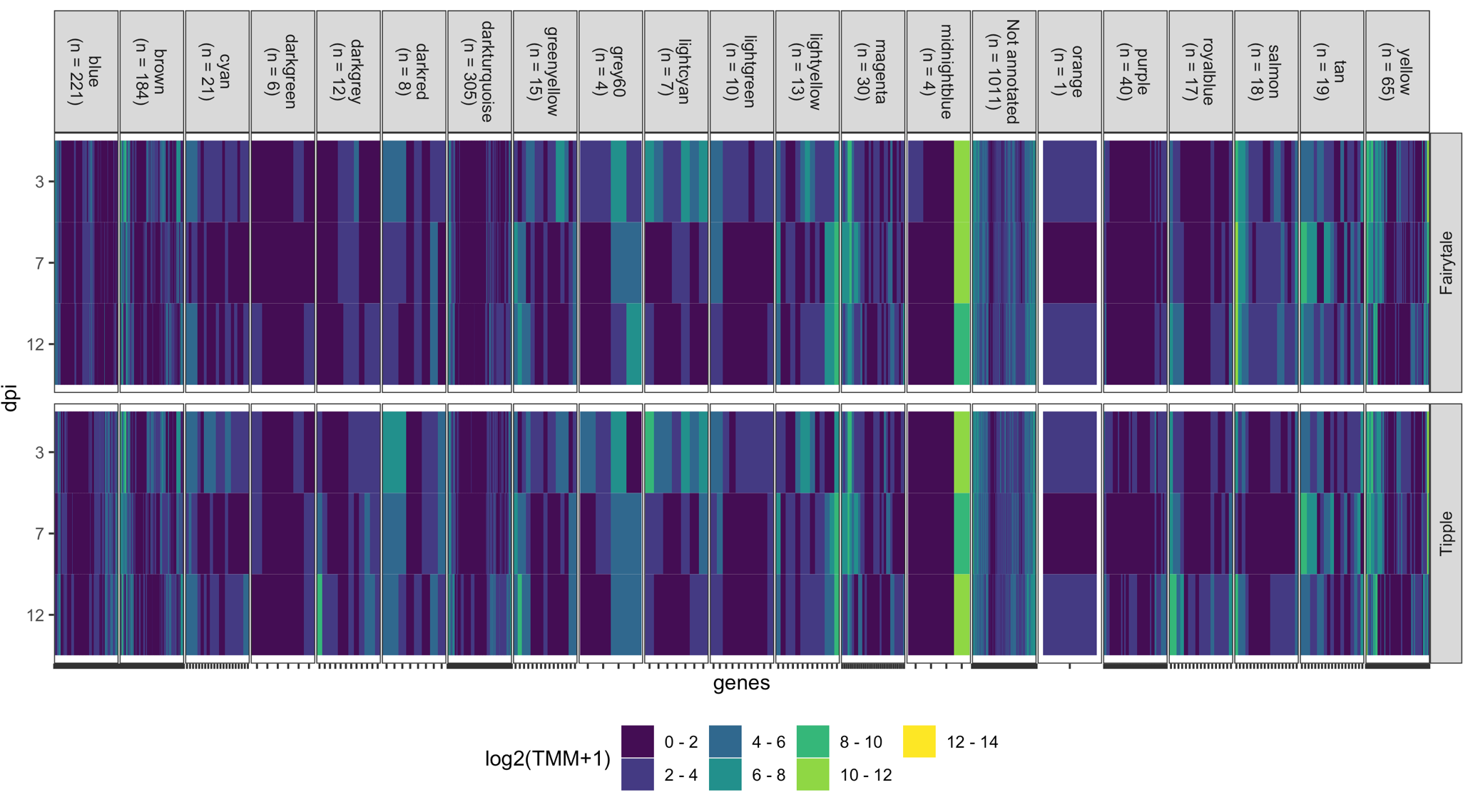


Supplementary Figure 14 Differentially expressed barley genes between Tipple and Fairytale control samples. Expression of genes which are higher expressed in one or the other barley variety sorted by their co-expression module membership. Abbreviations: TMM – Trimmed mean of M-values, n = Number of genes in the module.


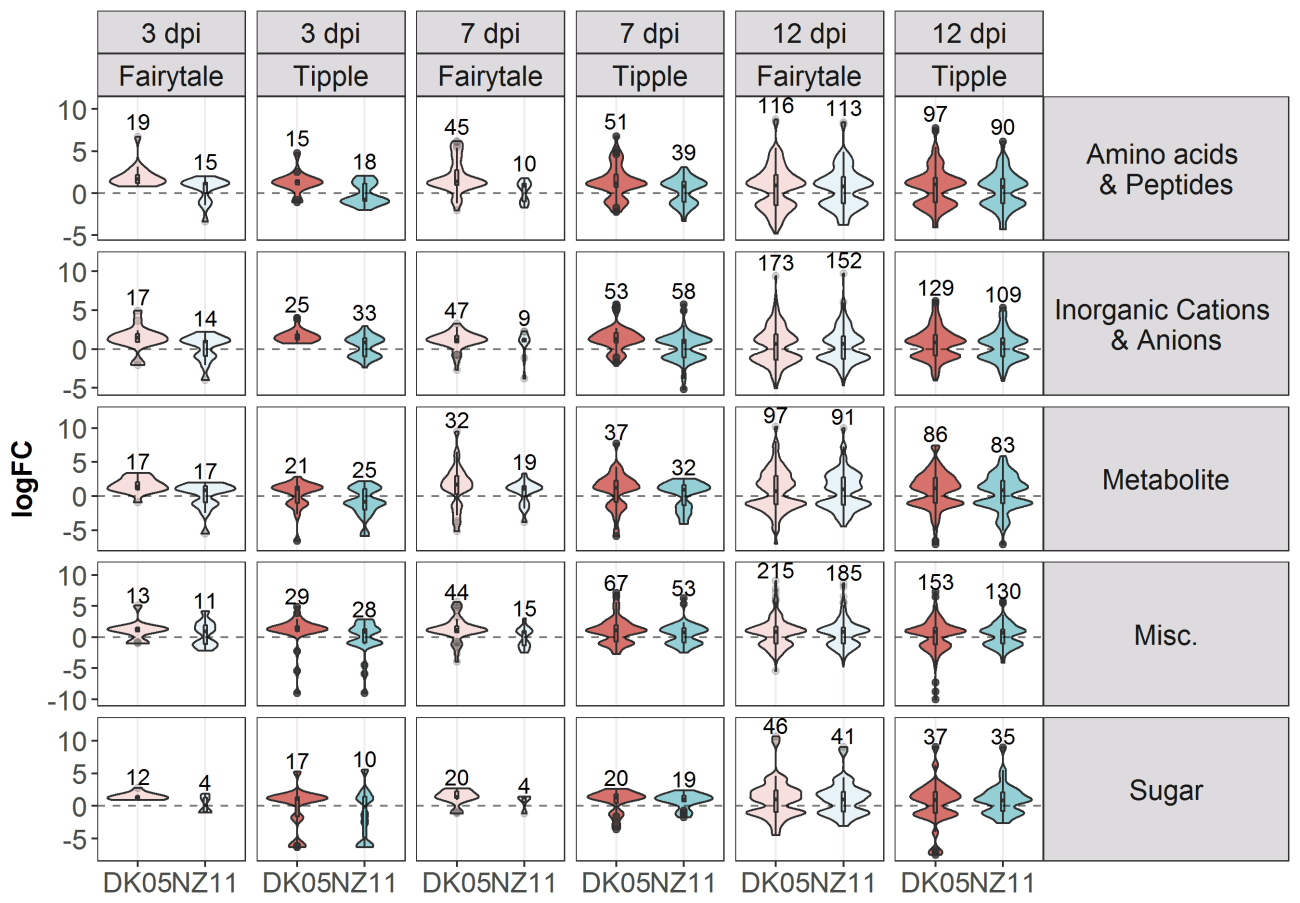


Supplementary Figure 15 Differentially expressed (DE) barley genes across different sub-categories of TRANSMEMBRANE TRANSPORTERS during Ramularia leaf spot (RLS) disease progression. Violin plots with included box-whisker plots depicting the general trend of up and down regulation during RLS progression on barley (Hordeum vulgare L) cv. Fairytale and cv. Tipple during foliar infection with Rcc isolates DK05 and NZ11.


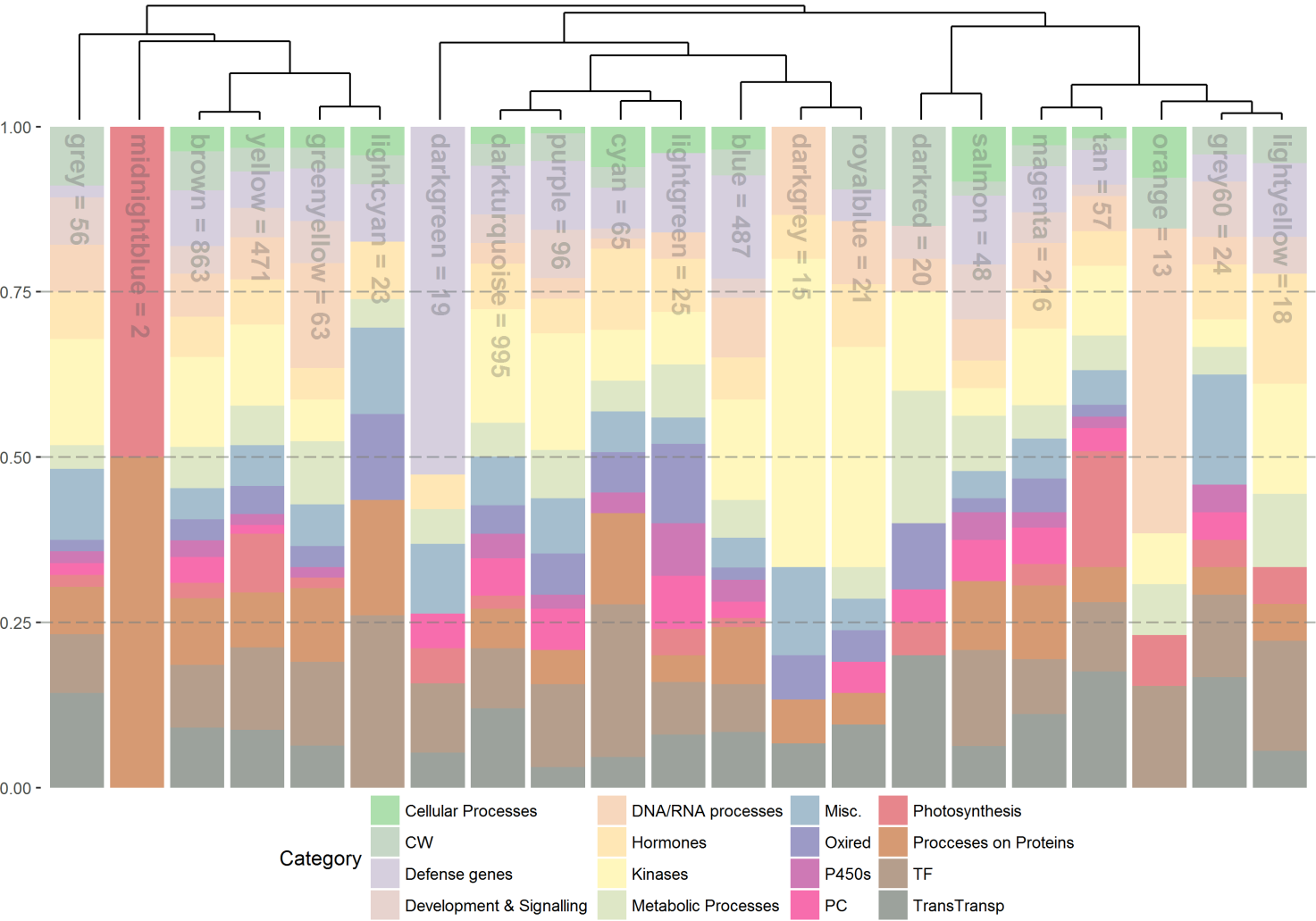


Supplementary Figure 16 Stacked relative bar plots depicting the proportions of categorized gene groups of the identified gene co-expression modules. Genes categorized in the group of ‘Not categorized’ genes were removed. Gene modules and associated bars are sorted by a hierarchical clustering represent by the dendrogramm. Name of module is presented inside the bar. The number corresponds to the amount of genes left inside the module after removing the category ‘Not categorized’.


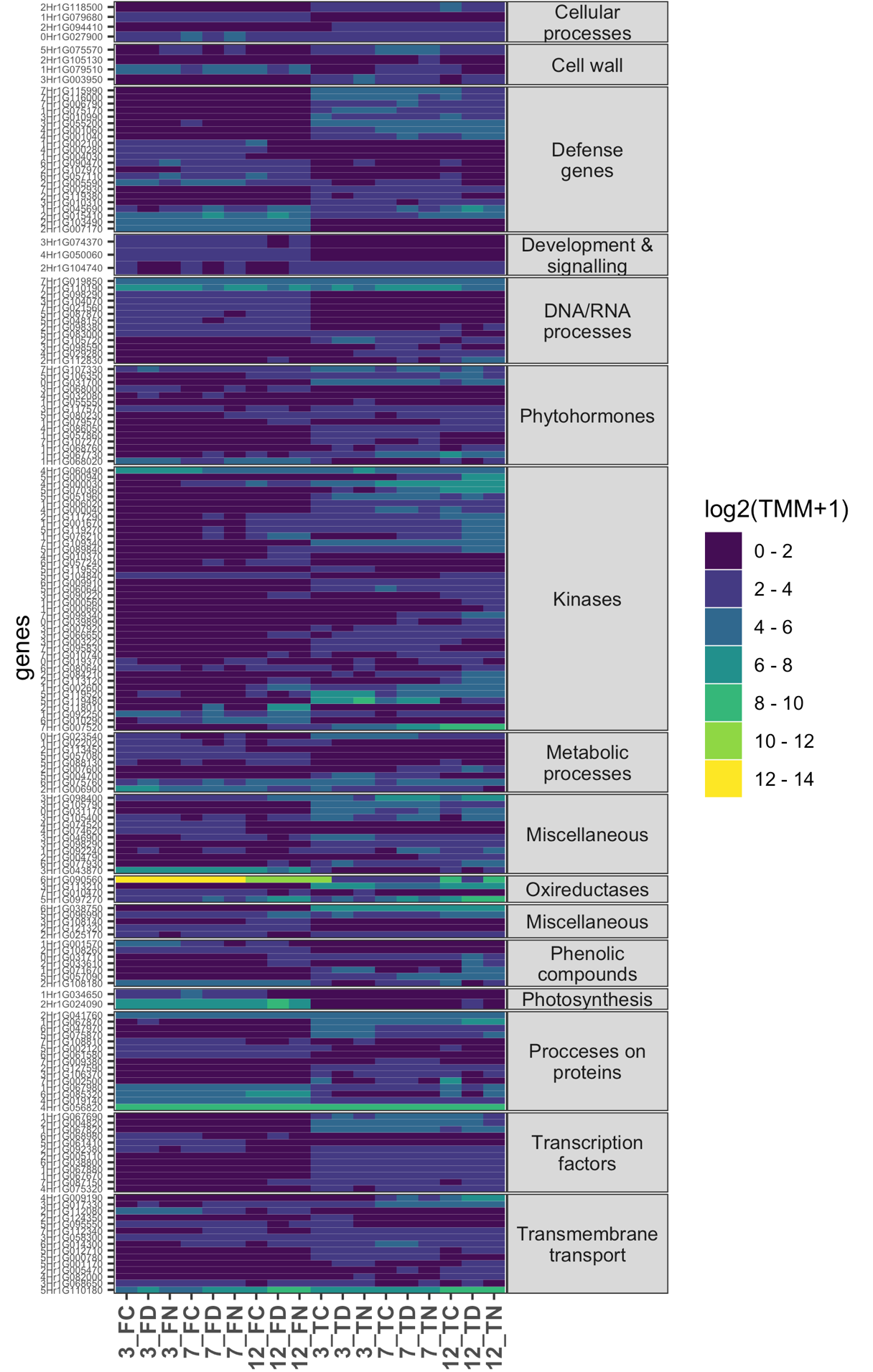


Supplementary Figure 17 Expression levels of genes in Co-expression subgroup VI. Genes in the *blue*, *darkgrey* and *royalblue* co-expression modules. Genes shown here have a TMM value of 5 in atleast 3 samples and genes that were assigned to Category “Not categorized” were removed. Abbreviations: F – cv Fairytale; T – cv Tipple, D – Rcc DK05, N – Rcc NZ11, TMM – Trimmed mean of M-values.


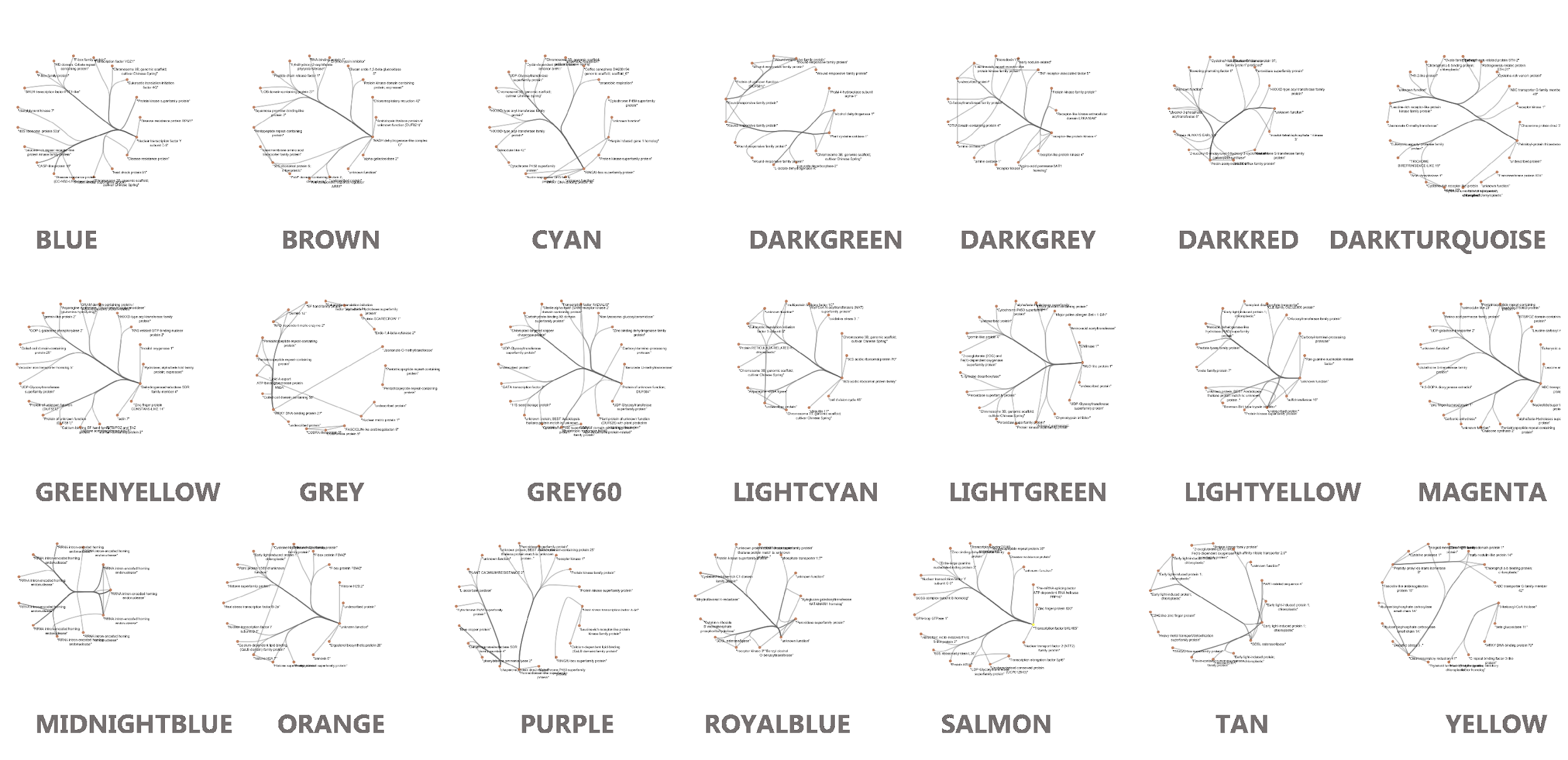


Supplementary Figure 18 Co-expression networks of 20 highest connected genes. Shown are the 20 genes with the strongest connection for each of the identified co-expression modules.


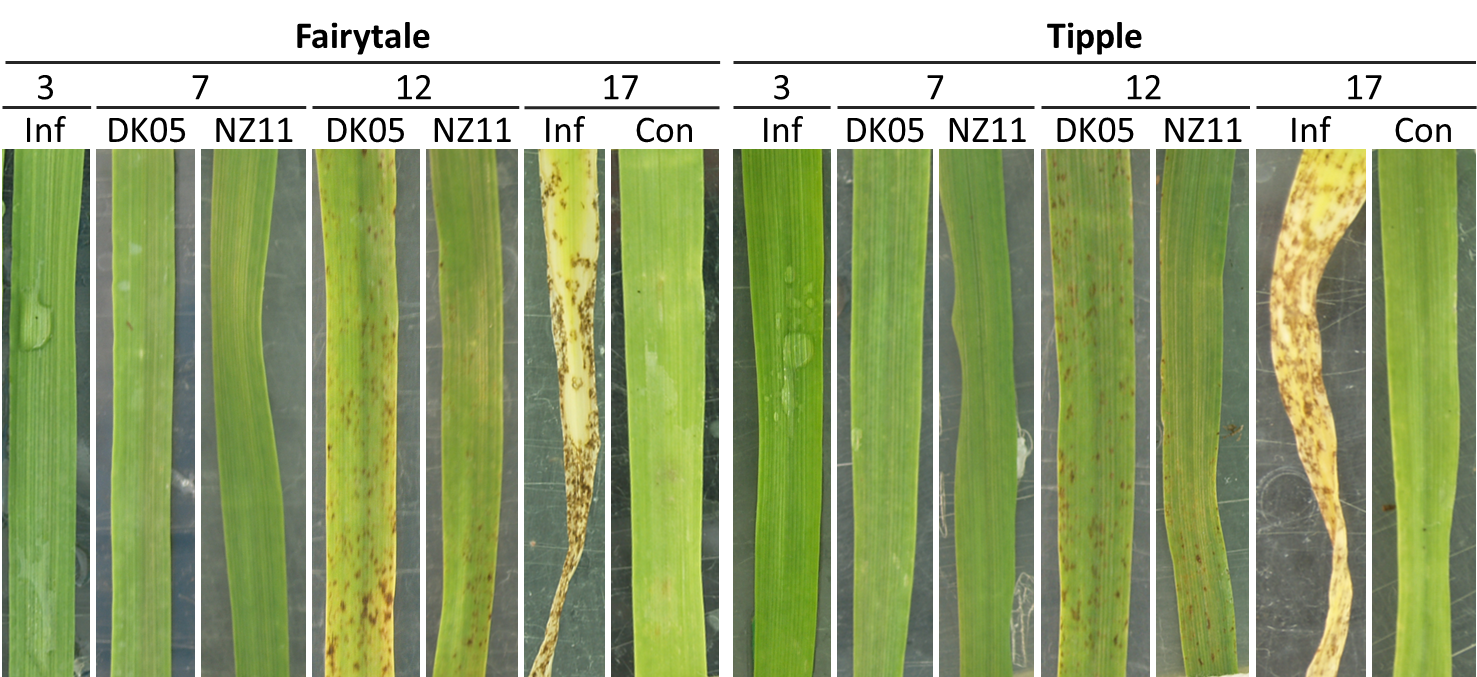


Supplementary Figure 19 Progress of necrotic leaf spot development at 3, 7, 12 and 17 days post inoculation (dpi) on barley cultivars Fairytale and Tipple infected with either DK05 or NZ11. Please note, this is an increased version of Figure 1.

Supplementary Figure SEQ SUpplementary_Figure \* ARABIC 18 Gene networks for top 20 connections between genes. Each network depicts one module identified in the WGCNA analysis and contains the top 20 connections between genes within the module. Modules *blue*, *cyan*, *darkgreen*, *darkgrey*, *lightgreen* and *magenta* also shown in Figure 5.
